# A Multifunctional 3D Supermolecular Co Coordination Polymer With Potential for CO_2_ Adsorption, Antibacterial Activity, and Selective Sensing of Fe^3+^/Cr^3+^ Ions and TNP

**DOI:** 10.3389/fchem.2021.678993

**Published:** 2021-07-15

**Authors:** Xiaojing Zhou, Lili Liu, Hang Kou, Shimei Zheng, Mingjun Song, Jitao Lu, Xishi Tai

**Affiliations:** School of Chemical and Chemical Engineering and Environmental Engineering, Weifang University, Weifang, China

**Keywords:** sensing, Fe^3+^/Cr^3+^, TNP, antibacterial, CO_2_ adsorption, magnetism, coordination polymer

## Abstract

A 3D supermolecular structure [Co_3_(L)_2_ (2,2′-bipy)_2_](DMF)_3_(H_2_O)_3_ 1) (H_3_L = 4,4′,4″-nitrilotribenzoic acid) has been constructed based on H_3_L, and 2,2′-bipy ligands under solvothermal conditions. Compound **1** can be described as a (3, 6)-connected kgd topology with a Schläfli symbol (4^3^)_2_(4^6^.6^6^.8^3^) formed by [Co_3_(CO_2_)_6_] secondary building units. The adsorption properties of the activated sample 1a has been studied; the result shows that 1a has a high adsorption ability: the CO_2_ uptakes were 74 cm^3^·g^−1^ at 273 K, 50 cm^3^·g^−1^ at 298 K, the isosteric heat of adsorption (Q_st_) is 25.5 kJ mol^−1^ at zero loading, and the N_2_ adsorption at 77 K, 1 bar is 307 cm^3^ g^−1^. Magnetic measurements showed the existence of an antiferromagnetic exchange interaction in compound **1**, besides compound **1** exhibits effective luminescent performance for Fe^3+^/Cr^3+^ and TNP.

## Introduction

Nowadays, the rapid detection of toxic organic and heavy metal ion pollutants has attracted more and more attention due to their harmful effects on the environment and human life (Rasheed and Nabeel, 2019; Haldar et al., 2020). For instance, nitroaromatic explosives (NACs), which include nitrobenzene, 2,4,6-trinitrophenol (TNP), 2-nitrotoluene, 2,4-dinitrotoluene, nitrobenzene, 4-nitrotoluene and 3-nitrotoluene, have many application in the chemical industry and can cause terrorism and environmental issues. Among NACs, TNP is highly toxic, it harms the microorganisms and the human body (Wollin and Dieter, 2005). Likewise, heavy metal pollutants are not degradable and tend to accumulate in ecosystems, imposing a threat to human beings because of their toxicity and carcinogenicity (Jia et al., 2017; Peng et al., 2018; Ashraf et al., 2019; Cai et al., 2019). Fe^3+^ is an abundant and essential transition metal for biological organisms, and plays an important role in biological processes, such as enzymatic reactions, nitrogen fixation in nitrogenases, and oxygen transport. It is also well known that inadequate or excess iron concentration can induce serious health problems including anemia, Alzheimer's disease, liver and kidney damage, diabetes and heart disease, mitochondrial DNA damage (Harigae, 2018; VanderMeulen and Sholzberg, 2018; Wallace et al., 2018; Sahoo and Crisponi, 2019; Fan et al., 2020). Similarly, Cr^3+^ has mutagenicity and cytogenetic toxicity, the scarcity or excess uptake of Cr^3+^ results in cardiovascular diseases and diabetes, mutations or malignant cells (Paul et al., 2015; Zhang et al., 2015; Dong et al., 2016; Rasheed and Nabeel, 2019), so it is urgent and necessary to detect metal ion pollutants in solution for the human security and environmental protection.

Various techniques have been developed to detect Fe^3+^/Cr^3+^ and TNP (Chen et al., 2018; Pavlačka et al., 2016; Sadak et al., 2017; Tian et al., 2017; Goswami et al., 2013; Wen et al., 2002); among them, fluorescence analysis has been very popular due to its simplicity, sensitivity, fast response, economical way, low interference (Carter et al., 2014; Guo et al., 2014). Therefore the development of excellent fluorescence sensors for the sensitivity of Fe^3+^/Cr^3+^ and TNP has become a focus. The use of coordination polymers for fluorescence analysis has been explored extensively (Zhang et al., 2018; Hu et al., 2014; Yi et al., 2016). The coordination polymers, built up from organic ligands and metal ions or clusters, are porous materials suitable for various applications including luminescence, magnetism, gas adsorption and separation, as well as catalysis, drug delivery, and proton conduction (Kurmoo, 2009; Huxford et al., 2010; Sun et al., 2013; Yamada et al., 2013; Li et al., 2014; Liu et al., 2014; Zhou. and Kitagawa, 2014; Chughtai et al., 2015; Lustig et al., 2017; Espallargas and Coronado, 2018).

Over the past few years, many luminescent coordiantion polymers have been synthesized to detect metal ions, anions, pH value, small molecules, gases and vapors (Kurmoo, 2009; Lan et al., 2009; Li et al., 2013; Ma et al., 2013; Zhang et al., 2015; Yu et al., 2017; Mi et al., 2019; Tang et al., 2020), in this contribution, we select a tricarboxytriphenylamine (H_3_L) as a ligand to construct a new Co coordiantion polymer is mainly based on the following considerations: 1) The conjugated and photoactive triphenylamine moiety makes the MOFs highly fluorogenic; 2) Lewis base N sites on the internal surface of the framework can improve the sensing of ions and adsorption of CO_2_; 3) The carboxylate groups have multiple coordination modes to coordinate the metal ions, and the uncoordinated O atoms can provide interaction sites for the metal ions and NACs (specifically, TNP containing three NO_2_ groups). Meanwhile, among the MOF sensors, highly economical and abundant Co. ions with magnetic properties have rarely been studied as sensors, mainly because the non-d^10^ electronic structures have low emission performance owing to d–d transitions (Mishra et al., 2014; Chen et al., 2017; Zhang et al., 2018; Zhao et al., 2018).

For the recent years, significant progress has been expended on the development of materials for CO_2_ capture, because CO_2_ is responsible for the global warming. Utilizing the activated carbon, zeolites or amine solutions for absorbing CO_2_ are considered the most adequate adsorbents, though the insufficient uptake capacity and high expense prevent these materials mass production (Zhang et al., 2014).

Many human diseases and infections are caused by unsafe drinking water and food containing bacteria such as *Escherichia coli*, *Staphylococcus aureus.* As to the low molecular weight antibacterial materials, they have many disadvantages, such as toxicity to the environment, short-term antibacterial activity. Hence, there is an urgent need for the development of effective antibacterial materials (Haendel et al., 2014; Kaur et al., 2020; Saira et al., 2020).

Taking the luminescence properties, CO_2_ adsorption and antibacterial activity into consideration, we used the coordination polymer as the multifunctional material for sensitivity as well as CO_2_ adsorption and antibacterial activity. In the manuscript, we obtained a Co. based coordiantion polymer [Co_3_(L)_2_ (2,2′-bipy)_2_](DMF)_3_(H_2_O)_3_ (denoted as compound **1**) under solvothermal conditions which has been utilized as a multifunctional MOF with preferential CO_2_ adsorption, antibacterial activity, selective sensing of metal ions (Fe^3+^, Cr^3+^) and TNP, meanwhile, magnetic measurements show that there exists an antiferromagnetic exchange interaction in compound **1**.

## Materials and Methods

### Synthesis of Compound 1

Co.(NO_3_)_2_•6H_2_O (29.1 mg), H_3_L (18.8 mg), and 2,2′-bipy (15.6 mg), *N*,*N*-dimethylformamide (3 ml), distilled water (1 ml), and ethanol (1 ml) were mixed in a 15 ml Teflon-lined stainless steel autoclave and heated at 100 °C for 72 h. Upon cooling at room temperature, purple crystals were prepared, which were washed with DMF and dried at 60°C for 6 h. Yield 38% (based on H_3_L), IR (KBr 4000–400 cm^−1^) 3463 (w), 3082 (w), 2,793 (w), 2,496 (w), 1,593 (s), 1,388 (s), 1,191 (w), 1,036 (w), 803 (m), 768 (m), 704 (m), 636 (w), 485 (m). Elemental analysis (%): Calcd for: C_71_H_67_Co_3_N_9_O_18_: C 56.39, H 4.43, N 8.34; Found: C 56.41, H 4.29, N 8.37.

## Results and Discussion

### Crystal Structure of Compound 1

**FIGURE 1:**
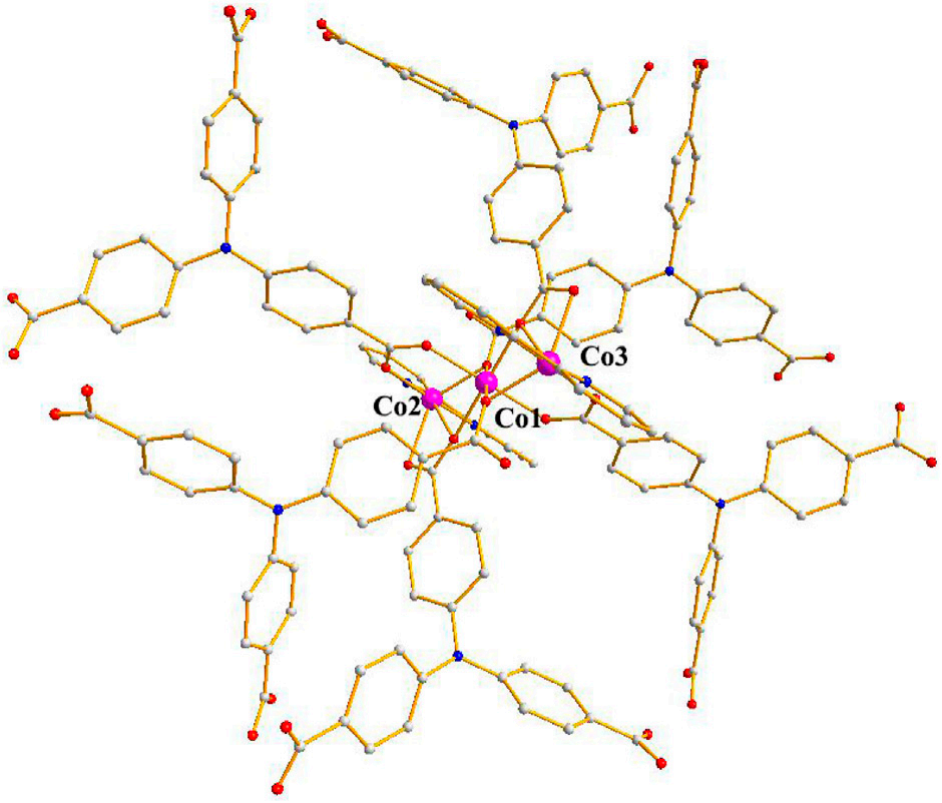
The coordination mode of Co atoms in compound **1** (C: Gray, N: Blue, Co:Purple, O: Red).

**FIGURE 2:**
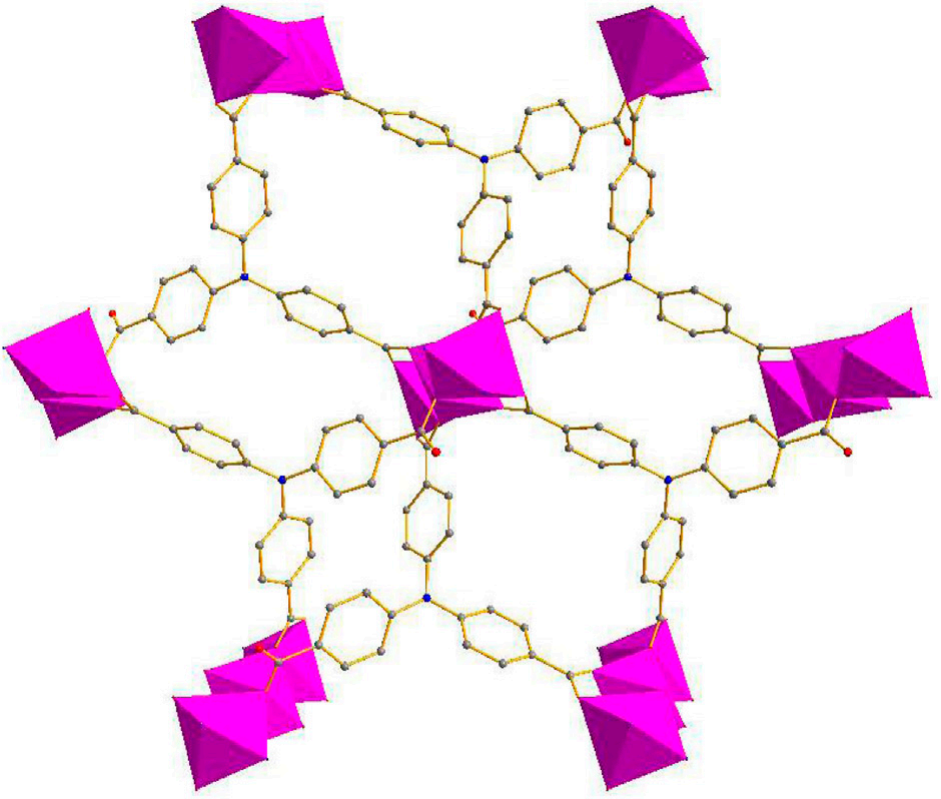
The 2D framework of compound **1** (Co:Purple, O: Red, N: Blue, C: Gray).

**FIGURE 3:**
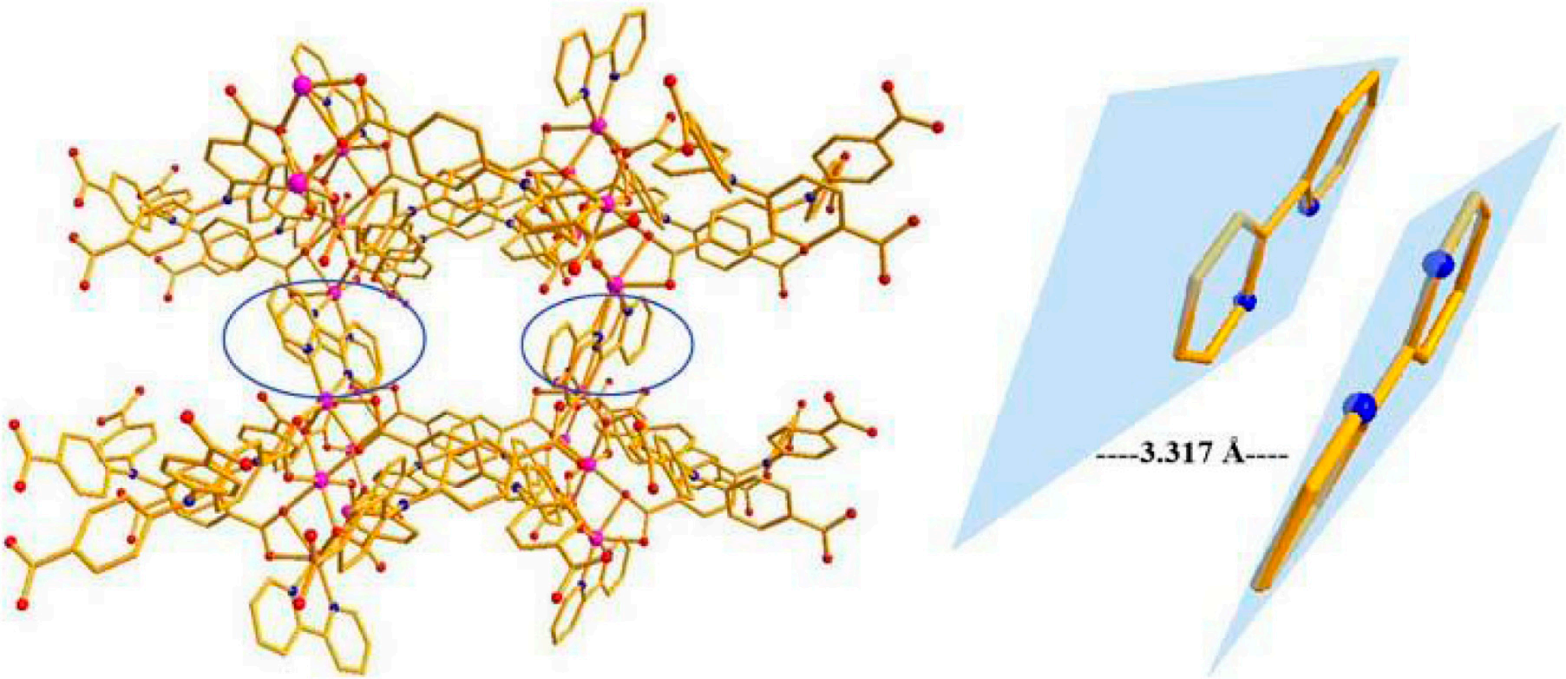
The structure of compound **1** and interactions of π–π stacking (C: Gray, O: Red, N: Blue, Co:Purple).

The single-crystal X-ray data were collected using the X-ray diffraction technique and the results showed that crystallization of compound 1 in the monoclinic space group *C*2*/c* and the presence of two independent Co. atoms, one 2,2′-bipy molecule, and one linker (L^3−^) in the asymmetric unit of 1 (Supplementary Figure S1). The Co1 atom is involved in coordination with six O atoms of the carboxylates of 6 L^3−^ ligands, showing an octahedral configuration (Co–O = 2.053–2.108 Å), the six-coordinated Co2 or its symmetry-related Co3 atom displays a distorted octahedral configuration, which is bonded with four carboxylate O atoms from 3 L^3−^ ligands, and 2 N atoms from one 2,2′-bipy molecule, Co2–O and Co2–N or Co3–O and Co3–N are in the range of 2.007–2.183 Å and 2.085–2.115 Å, respectively (Figure 1) (Mistri et al., 2017; Zhou et al., 2019), the bonding mode of the carboxylate toward the six Co^2+^ ions is μ^6^-η^1^:η^2^:η^1^:η^1^:η^2^:η^0^ (Supplementary Scheme 1 in the Supplementary Information), the adjacent Co1–Co3 atoms (Co···Co. separation, 3.168–6.337 Å) are united together by six carboxylates to form trinuclear Co.(II) clusters, each cluster can be connected to the adjacent ones to evolve a unique bylayer 2D framework, which contains quadrangle grids with a size of 7.0 Å × 6.4 Å running along the b-axis (atom-to-atom distance) (Figure 2). The 2D layers are then further converted into a 3D (supramolecular) structure via interactions of π–π stacking with the distance of 3.317 Å (Figure 3). The solvent-accessible volume in compound 1 was found to be 28.8 % (calculated using the PLATON software.12 after removal of solvent molecules).

If the L^3−^ ligands are defined as three-connected nodes, and the trinuclear Co.(II) cluster as six-connected nodes, the entire structure of Co. coordiantion polymer can be denoted as a (3, 6)-connected two-nodal net with a point symbol of {4^3^}_2_{4^6^.6^6^.8^3^}, displaying the kgd topology (Supplementary Figure S2) (Kim et al., 2012; Wang et al., 2014).

### Gas Sorption Experiment

**FIGURE 4:**
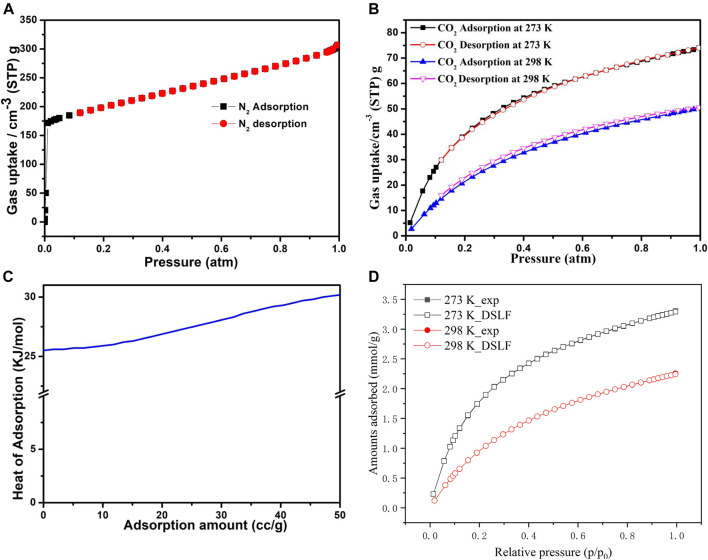
**(A) N**_**2**_ adsorption isotherms of 1a at 77 K; **(B)** CO_2_ adsorption isotherms of **1a** at 273 K, 298 K; **(C)** Heat of CO_2_ adsorption as a function of uptake amount; **(D)** The DSLF model for CO_2_ adsorption isotherms.

The as-synthesized compound **1** was subjected to stirring with methanol at ambient temperature for 24 h to remove the solvent in the pores, followed by filtration of the compound and keeping at 60°C for 6 h in an oven. The compound was then heated for 24 h at 100°C under a vacuum to obtain the activated sample, 1a. The N_2_ adsorption isotherm was acquired at 77 K, the result indicates that 1a displays a reversible type-I adsorption isotherm with the Brunauer–Emmett–Teller (BET) surface area of 658 m^2^·g^−1^, and the N_2_ uptake (1 atm) reached 307 cm^3^·g^−1^ (Figure 4A). Meanwhile, the CO_2_ adsorption isotherms for 1a were measured at 273 and 298 K; at 273 K (1 atm), and 298 K (1 atm) the CO_2_ uptakes reached a maximum of 74 cm^3^·g^−1^ and 50 cm^3^·g^−1^, respectively (Figure 4B). The PXRD pattern of compound 1a remained stable after the adsorption of N_2_ and CO_2_ (Supplementary Figure S11). Considering the adsorption isotherm at 298 K, the observed CO_2_ adsorption capacity of 1a is better than the metal-organic frameworks including [Zn(BPTC)_0.5_ (Tz)]•DMF•CH_3_OH, JUC-MOF56, {[Cd_2_ (tdz)_2_ (4,4′-bpy)_2_]•6.5H_2_O}n, [Zn_2_ (TCA) (BIB)_2.5_]•(NO_3_), ([Zr_6_O_4_(OH)_8_(H_2_O)_4_(BTEB)_2_], and {[Cd_4_ (hbhdpy)_2_ (bdc-NH_2_)_3_ (DMA)_2_]•(H_2_O)_4_}n that are summarized in Supplementary Table S3 (Hong et al., 2017; Kong et al., 2018; Yao et al., 2018; Zhou et al., 2018; Liu et al., 2019). The adsorption isotherms show typical type-I sorption isotherm with fast kinetics and good reversibility, further indicating its microporosity.

To understand better the CO_2_ adsorption, we calculated the Q_st_ (isosteric heat) for 1a using the CO_2_ adsorption data, which were recorded at 273 and 298 K using the virial coefficient method. As depicted in Figure 4C, the Q_st_ value reached 25.5 kJ mol^−1^ at zero loading, showing the good interactions of framewok–CO_2_ in compound **1**, which can be ascribed to the uncoordinated O sites, N-donor of the H_3_L, and the unique microporous channels. The dual-site Langmuir-Freundlich (DSLF) model was also utilized to fit the absolute adsorption isotherms of CO_2_ from molecular simulations (Figure 4D). The result shows that the simulated CO_2_ adsorption isotherms are in accordance with the experimental datas.

### Magnetic Properties

**FIGURE 5:**
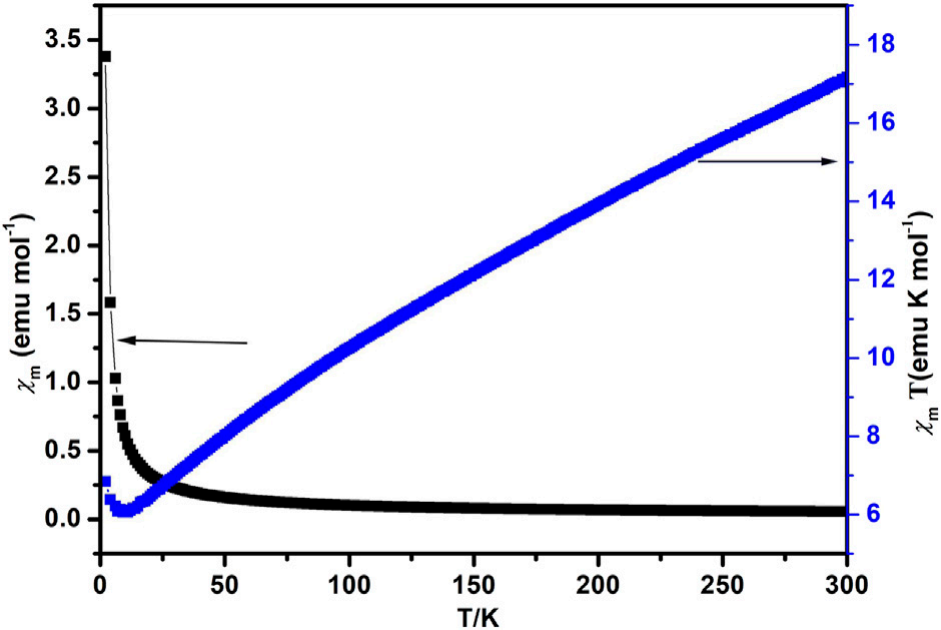
Magnetic susceptibility of compound **1** plotted as *χ*_m_ vs. T (black) and *χ*_m_T vs. T curves (blue).

Compound **1** was subjected to magnetic susceptibility measurements in the range 2–300 K at 1,000 Oe field, plots of the variable temperature magnetic susceptibility for compound **1** in the form of *χ*_m_T vs T are presented in Figure 5. Compound **1** showed a higher *χ*_m_T of about 17.17 emuK·mol^−1^ for a Co_3_ unit at 300 K than the calculated spin-only value for three isolated Co^2+^ ions (5.75 emuK mol^−1^ and S = 3/2), and lies well in the range identified for octahedral Co^2+^ ions in the ^4^T_2g_ state, which is due to the significant contribution of orbitals belonging to Co^2+^ ion in the octahedral surroundings. Upon cooling, the *χ*_m_T value decreases sharply until the temperature descends to 11 K, then it starts to increase rapidly, attaining a minimum value of 6.84 emuK·mol^−1^ at 2 K. The behavior is consistent with antiferromagnetic phenomenon between 11–300 K. The magnetic susceptibility fits the Curie−Weiss law well above 130 K, giving C = 20.95 emuK·mol^−1^ and *θ* = −185.5 K, indicating an antiferromagnetic interaction between the Co_3_ units.

### Luminescent Emission

The solid-state emission spectra of compound **1** and H_3_L ligand are depicted in Supplementary Figure S3. The free ligand H_3_L displayed emission at 448 nm when it is excited at 330 nm. Meanwhile, compound **1** showed an emission peak at 420 nm under excitation at 345 nm, there is a blue shift of 28 nm in comparison with the H_3_L ligand. The fluorescence emission of compound **1** can be associated with the corresponding intraligand transitions (π* → π and π* → n) (Zhang et al., 2018).

**FIGURE 6:**
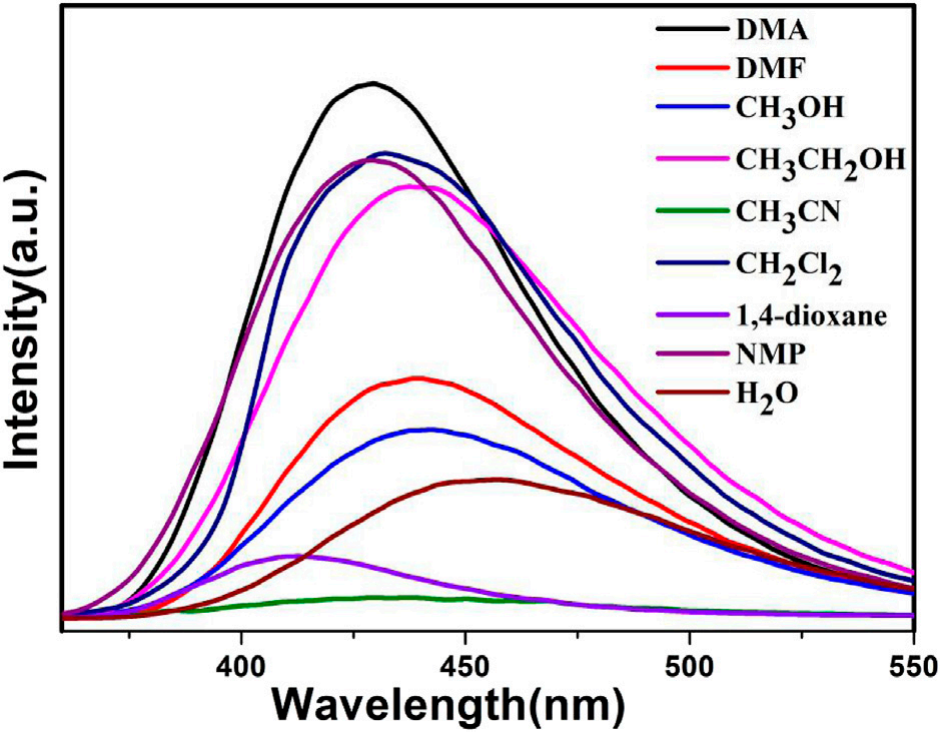
Fluorescent spectra of compound **1a** in different solvents.

We select compound **1a** as a representative example to study its sensing sensitivity. Dispersions of compound **1a** (3 mg) in different solvents, namely DMA, DMF, methanol, ethanol, acetonitrile, dichloromethane, 1,4-dioxane, NMP (*N*-methyl-2-pyrrolidone), and H_2_O (3 ml) were prepared, and the emission spectra were measured. As shown in Figure 6, the luminescence intensity was affected by the solvent, especially for DMA.

**FIGURE 7:**
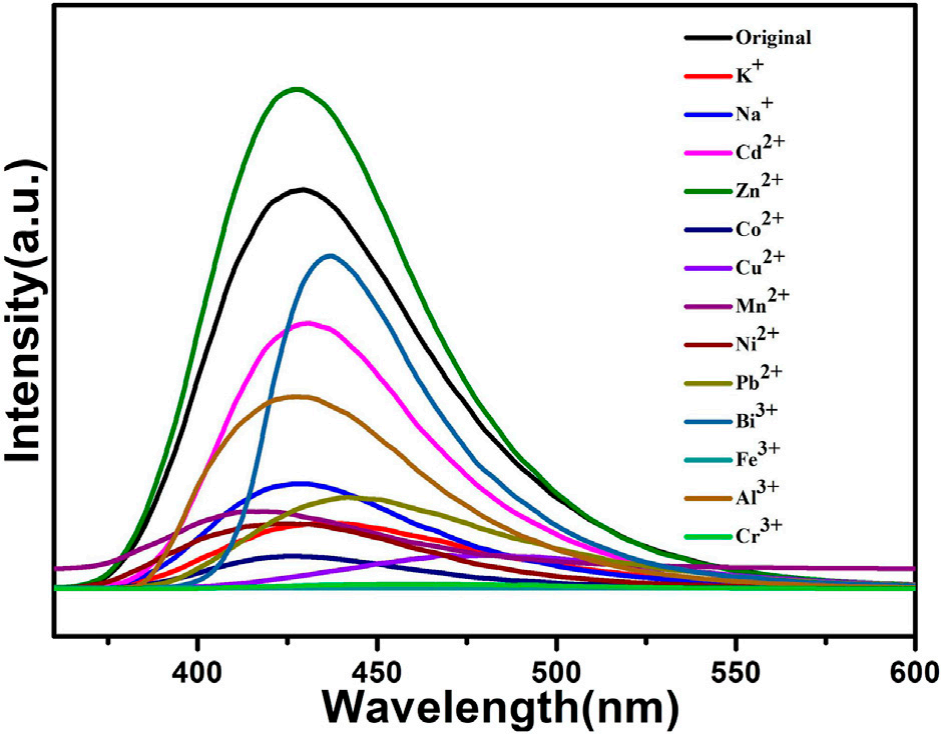
Fluorescent analysis of 1a toward various metal ions (10^–2^ M) in DMA solution.

**FIGURE 8:**
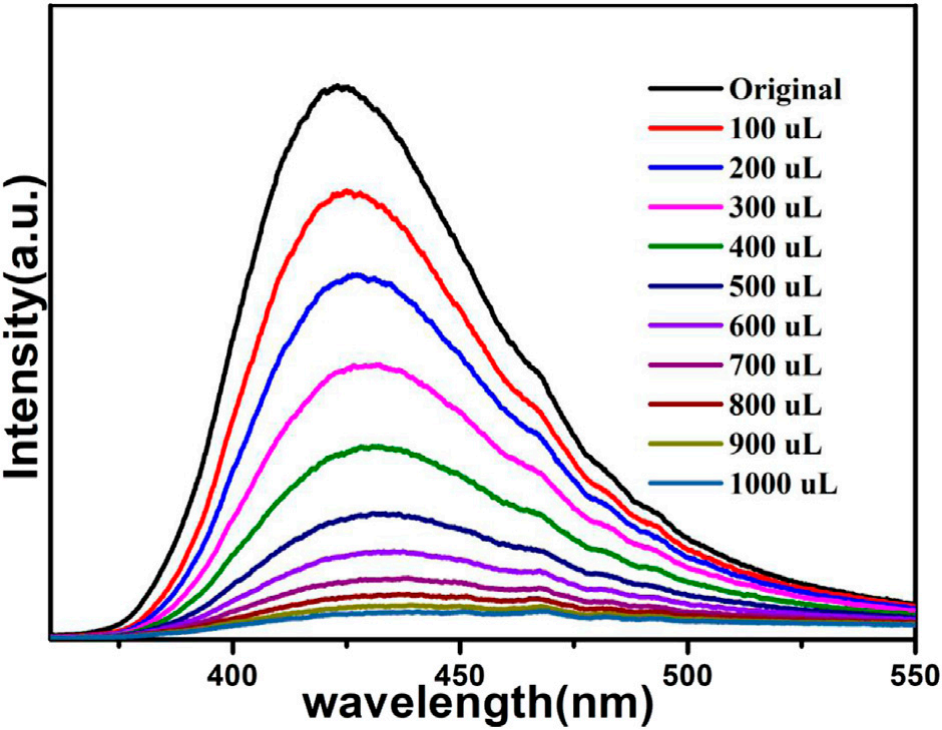
Fluorescence of 1a in DMA containing different volumes of Fe^3+^ (1 × 10^–3^ M).

**FIGURE 9:**
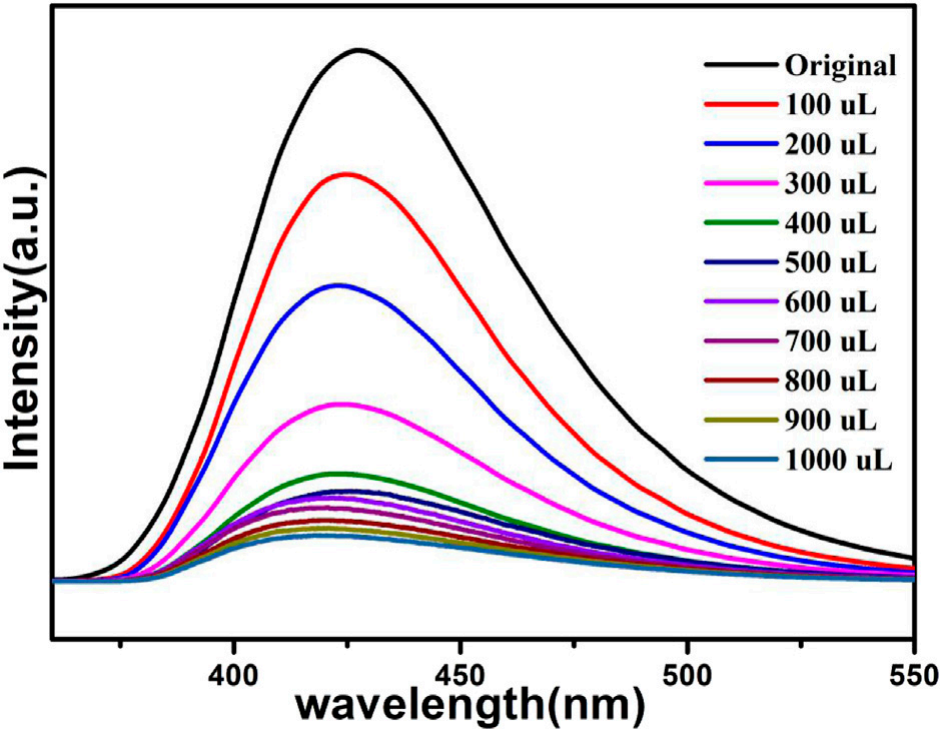
Fluorescence of 1a in DMA containing different volumes of Cr^3+^ (5 × 10^–3^ M).

The above fluorescence performance prompted us to explore their potential sensing of metal ions. Samples of grounded 1a were dispersed in M(NO_3_)_x_ DMA solution separately (3 mg each sample in 3 ml, 0.01 M, M(NO_3_)_x_) (M = K^+^, Cd^2+^, Na^+^, Zn^2+^, Co^2+^, Cu^2+^, Mn^2+^, Ni^2+^, Pb^2+^, Bi^3+^, Fe^3+^, Al^3+^, Cr^3+^), followed by ultrasonication for 1 h to obtain the uniform suspensions, the luminescence intensities of the suspensions were measured. The different emission peaks are shown in Figure 7, the metal ions exhibited different influence on the luminescence intensity, and the result showed that Fe^3+^ and Cr^3+^ exhibited a remarkable effect to quench the luminescence of **1a,** which indicate the high sensitivity performance of 1a towards Fe^3+^ and Cr^3+^, the PXRD of compound 1a were measured after sensing the metal ions which remained their structural integrity (Supplementary Figure S12). Furthermore, the anti-interference experiments were performed and the results indicated that the presence of other metal ions would not disturb the selective sensing of Fe^3+^ or Cr^3+^ (Supplementary Figure S7). Meanwhile, 3 mg samples of 1a were ground and immersed in DMA solution, sonicated for 1 h, the well-dispersed original suspensions were obtained, the Fe^3+^ or Cr^3+^ have been prepared in 1 × 10^–3^ M or 5 × 10^–3^ M DMA solution. The emission intensity decreased by gradually increasing the volume of Fe^3+^ and Cr^3+^ (Figures 8, 9). Compound 1a was centrifuged and washed by DMA solvent after sensing Fe^3+^ or Cr^3+^, the framework of the regenerated samples retained their stability, and reused for three cycles, the PXRD pattern of compound 1a is consistent with the recovered samples after three cycles (Supplementary Figure S13).

The fluorescence quenching efficiency can be discussed though the linear Stern–Volmer (S–V) equation: *I*_*0*_/*I* = 1 + *K*_sv_ [M], where I_0_ and I are the fluorescence intensities before and after the addition of Fe^3+^ or Cr^3+^, *K*_sv_ and [M] are the quenching constant and the concentration of Fe^3+^ or Cr^3+^, the Stern–Volmer analysis of quenching effect on Fe^3+^ and Cr^3+^ ions show that the values of *K*_sv_ for Fe^3+^ and Cr^3+^ ions are 5.4 × 10^4^ M^−1^, 7.83 × 10^3^ M^−1^, and the limit detection of Fe^3+^ and Cr^3+^ are 0.278 mM, 1.91 mM respectively (Figs. S4 and S5).

The results indicate that compound 1a has the potential to act as a luminescence sensor toward Fe^3+^, Cr^3+^.

**FIGURE 10:**
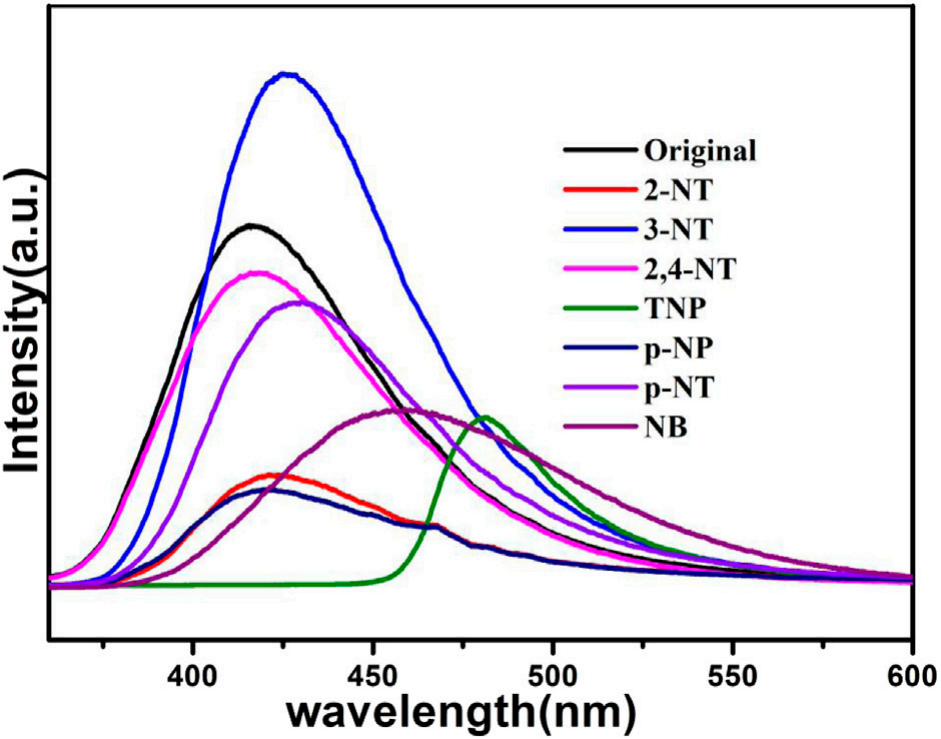
Fluorescence of 1a in NACs solutions (10^–3^ M).

The NACs are explosive and environmentally deleterious. They have been used a lot in the chemical industry, so it is necessary to develop effective and quick detection of NACs. As presented in Figure 10, the luminescent intensity of 1a is completely quenched at 425 nm in the presence of TNP, while no obvious luminescent changes of 1a can be observed in other NACs, confirming that TNP has a pronounced emission quenching of compound **1a**, while other NACs showed less pronounced quenching.

**FIGURE 11:**
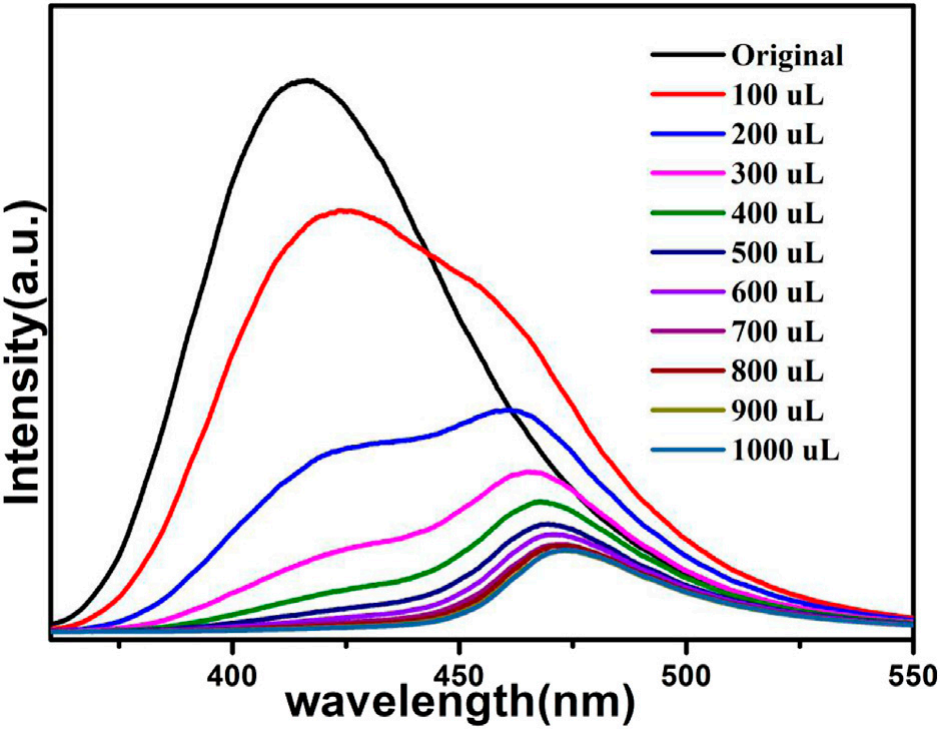
Fluorescence of 1a in DMA containing different volumes of TNP (10^–3^ M).

To investigate further the sensitivity of 1a for TNP, a fluorescence titration study of TNP was conducted (Figure 11), the results showed that with increased incorporation of TNP solution (10^–3^ M), the luminescent intensity drastically decreased. Moreover, For the emission band of 1a, there is a large bathochromic shift of 39 nm, which is due to the energy transfer between TNP and compound **1a** (Gogia and Mandal, 2019). The quenching effect of TNP on compound **1** can also be explained by the Stern-Volmer equation, and the details are provided in the SI. The S–V plot shows that the concentration of TNP and *I*_0_/*I* possess a direct relationship over the added TNP volume range (100–1000 μL), with a linear fit coefficient value of 0.982. It is commendable that the *K*_sv_ value of sensing TNP reaches 3.99 × 10^5^ M^−1^ (Supplementary Figure S6), which is one of the highest reported values for TNP sensing, and the limit detection of TNP is 0.0376 mM (Hong et al., 2017; Hua et al., 2018; Gogia and Mandal, 2019; Ghorai et al., 2019; Wang et al., 2019).

In addition, the Fe^3+^, Cr^3+^ and TNP solutions exhibit an absorption in the 260–500 nm range, which has overlaps with the excitation of compound 1a (Figs. S8 and S9). This shows the energy of excited light is taken by Fe^3+^, Cr^3+^ or TNP, so the transfer of energy from L^3-^ to Co^2+^ is blocked, resulting the quenching effect on compound 1a. The sensing mechanism for metal ions can be attributed to the suppression of luminescence resonance energy transfer and the enhancement of intermolecular electron transfer (Chen et al., 2018).

### Antibacterial Activity

The antibacterial activities of compound **1** against *Staphylococcus aureus* and *Escherichia coli* were measured using the transparent ring method. Compound **1**, the organic linker of TCA, and 2,2′-bipy were dissolved in distilled water with a concentration of 2 mg/ml. All the cultures were incubated for 18 h at 37 °C.

The results of the inhibition zone (ZOI) are shown in Table 1 which reveals the antibacterial potential of compound **1** against *E. Coli*, whereas compound **1** does not have antibacterial activity against *S. aureus* (Supplemetary Figure S16). Therefore, compound **1** has potential applications as an antibacterial agent.

**TABLE 1:** Inhibition zone diameters of compound **1** (A), H_3_L (B) and 2,2′-bipy (C).

Diameters	A	B	C
Samples of inhibition zone (mm)			
*Escherichia coli*	5	0	0
*Staphylococcus aureus*	0	0	0

## Conclusions

A new fluorescent 3D supramolecular Co. coordination polymer that contains uncoordinated O atoms in the channels was synthesized and characterized. The activated 1a exhibits a strong affinity toward CO_2_ molecules, with the adsorption of 74 cm^3^·g^−1^ (273 K, 1 atm). Magnetic measurements show that an antiferromagnetic exchange interaction exists in compound **1**. Moreover, compound 1 shows luminescence quenching with Fe^3+^/Cr^3+^ metal ions, further studies on detection of NACs showed high performance for sensing TNP. These results may contribute to the design of more multifunctional coordiantion polymers.

## Data Availability

The datasets presented in this study can be found in online repositories. The names of the repository/repositories and accession number(s) can be found below: Cambridge Crystallographic Data Centre (CCDC, https://www.ccdc.cam.ac.uk/), identification number 1961577
