# Supplementary material for: A Multifunctional 3D Supermolecular Co Coordination Polymer With Potential for CO2 Adsorption, Antibacterial Activity, and Selective Sensing of Fe3+/Cr3+ Ions and TNP
Source: Front Chem. 2021 Jul 15;9:678993. doi: 10.3389/fchem.2021.678993 (PMC8321245; doi:10.3389/fchem.2021.678993)

checkCIF/PLATON report

You have not supplied any structure factors. As a result the full set of tests cannot be run.

THIS REPORT IS FOR GUIDANCE ONLY. IF USED AS PART OF A REVIEW PROCEDURE FOR PUBLICATION, IT SHOULD NOT REPLACE THE EXPERTISE OF AN EXPERIENCED CRYSTALLOGRAPHIC REFEREE.

No syntax errors found. CIF dictionary Interpreting this report

Datablock: a_sq

Bond precision: C-C = 0.0026 A Wavelength=0.71073

Cell:	a=13.7148(9)	b=21.0489(13)	c=22.8914(14)
	alpha=90	beta=91.678(2)	gamma=90
Temperature:	273 K		

	Calculated	Reported
Volume	6605.5(7)	6605.5(7)
Space group	C 2/c	C 2/c
Hall group	-C 2yc	-C 2yc
Moiety formula	C62 H40 Co3 N6 O12 [+ solvent]	?
Sum formula	C62 H40 Co3 N6 O12 [+ solvent]	C62 H40 Co3 N6 O12
Mr	1237.79	1237.79
Dx,g cm-3	1.245	1.245
Z	4	4
Mu (mm-1)	0.804	0.804
F000	2524.0	2524.0
F000’	2529.20	
h,k,lmax	17,27,29	17,27,29
Nref	7693	7665
Tmin,Tmax	0.834,0.873	0.639,0.746
Tmin’	0.713	

Correction method= # Reported T Limits: Tmin=0.639 Tmax=0.746
AbsCorr = MULTI-SCAN

Data completeness= 0.996 Theta(max)= 27.635

R(reflections)= 0.0344(6039) wR2(reflections)= 0.0799(7665)

S = 1.031 Npar= 376

The following ALERTS were generated. Each ALERT has the format
test-name_ALERT_alert-type_alert-level.
Click on the hyperlinks for more details of the test.

Alert level B

```
PLAT430_ALERT_2_B Short Inter D...A Contact  O3        ..O6                2.71 Ang.
```

Alert level G

```
PLAT004_ALERT_5_G Polymeric Structure Found with Maximum Dimension          2 Info
PLAT199_ALERT_1_G Reported _cell_measurement_temperature ..... (K)        273 Check
PLAT200_ALERT_1_G Reported   _diffrn_ambient_temperature ..... (K)        273 Check
PLAT232_ALERT_2_G Hirshfeld Test Diff (M-X) Co1      --N1         .       7.1 s.u.
PLAT232_ALERT_2_G Hirshfeld Test Diff (M-X) Co1      --O3_f       .      40.1 s.u.
PLAT232_ALERT_2_G Hirshfeld Test Diff (M-X) Co1      --O4_f       .      12.8 s.u.
PLAT606_ALERT_4_G VERY LARGE Solvent Accessible VOID(S) in Structure        ! Info
PLAT793_ALERT_4_G Model has Chirality at C24         (Centro SPGR)          R Verify
PLAT869_ALERT_4_G ALERTS Related to the Use of SQUEEZE Suppressed           ! Info
```

---

0 **ALERT level A** = Most likely a serious problem - resolve or explain
1 **ALERT level B** = A potentially serious problem, consider carefully
0 **ALERT level C** = Check. Ensure it is not caused by an omission or oversight
9 **ALERT level G** = General information/check it is not something unexpected

2 ALERT type 1 CIF construction/syntax error, inconsistent or missing data
4 ALERT type 2 Indicator that the structure model may be wrong or deficient
0 ALERT type 3 Indicator that the structure quality may be low
3 ALERT type 4 Improvement, methodology, query or suggestion
1 ALERT type 5 Informative message, check

It is advisable to attempt to resolve as many as possible of the alerts in all categories. Often the minor alerts point to easily fixed oversights, errors and omissions in your CIF or refinement strategy, so attention to these fine details can be worthwhile. In order to resolve some of the more serious problems it may be necessary to carry out additional measurements or structure refinements. However, the purpose of your study may justify the reported deviations and the more serious of these should normally be commented upon in the discussion or experimental section of a paper or in the "special_details" fields of the CIF. checkCIF was carefully designed to identify outliers and unusual parameters, but every test has its limitations and alerts that are not important in a particular case may appear. Conversely, the absence of alerts does not guarantee there are no aspects of the results needing attention. It is up to the individual to critically assess their own results and, if necessary, seek expert advice.

Publication of your CIF in IUCr journals

A basic structural check has been run on your CIF. These basic checks will be run on all CIFs submitted for publication in IUCr journals (*Acta Crystallographica*, *Journal of Applied Crystallography*, *Journal of Synchrotron Radiation*); however, if you intend to submit to *Acta Crystallographica Section C* or *E* or *IUCrData*, you should make sure that full publication checks are run on the final version of your CIF prior to submission.

Publication of your CIF in other journals

Please refer to the *Notes for Authors* of the relevant journal for any special instructions relating to CIF submission.

PLATON version of 30/01/2018; check.def file version of 30/01/2018

Datablock a_sq - ellipsoid plot

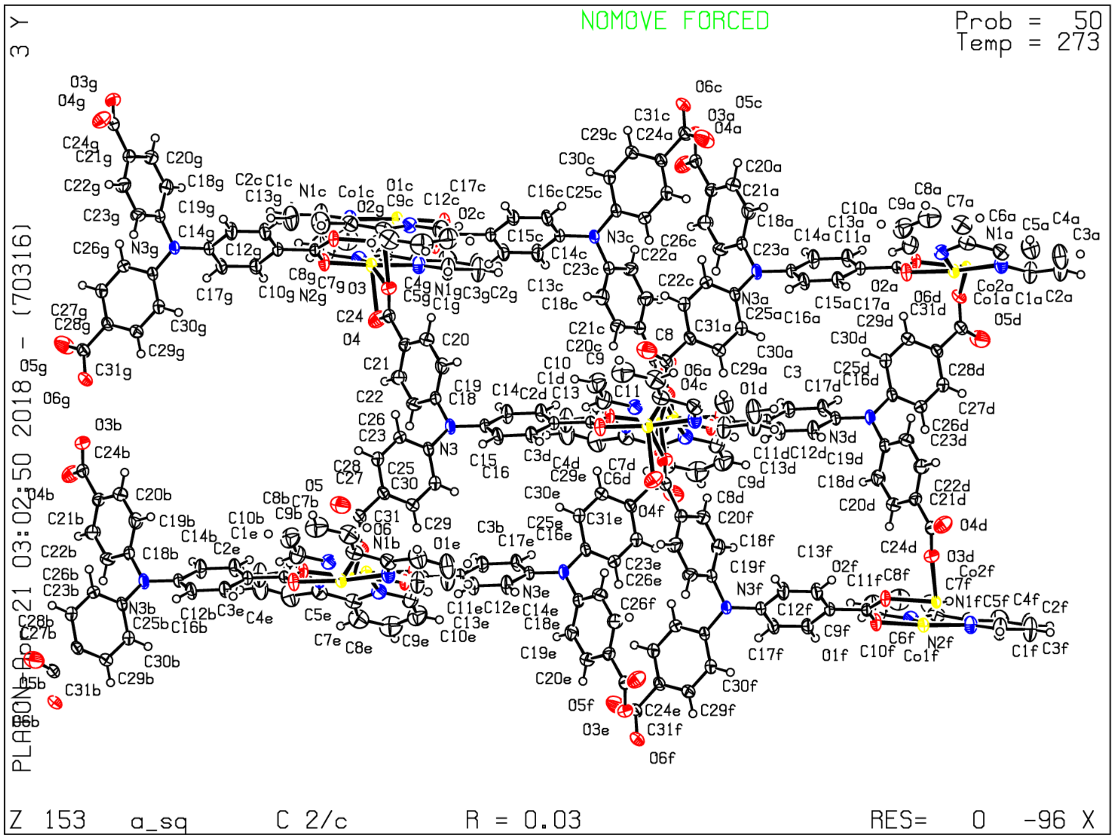

Supplement: Supplementary file 1 [file DataSheet1.PDF]
