# Supplementary material for: A Multifunctional 3D Supermolecular Co Coordination Polymer With Potential for CO2 Adsorption, Antibacterial Activity, and Selective Sensing of Fe3+/Cr3+ Ions and TNP
Source: Front Chem. 2021 Jul 15;9:678993. doi: 10.3389/fchem.2021.678993 (PMC8321245; doi:10.3389/fchem.2021.678993)
Supplement: Supplementary file 2 [file DataSheet2.doc]

**Electronic Supplementary Information (ESI) for:**

**A multifunctional 3D supermolecular Co-MOF with potential for CO_2_ adsorption, antibacterial activity, selective sensing Fe^3+^/Cr^3+^ ions and TNP**

**Xiaojing Zhou, Lili Liu, Hang Kou, Shimei Zheng, Mingjun Song, Jitao Lu,* Xishi Tai***

*School of chemical & Chemical Engineering and enviromental engineering ,Weifang University, Weifang, 261061, P. R China*

**Physical Measurements**

All chemicals were commercial available, used without further purification. FT-IR spectra were collected on a nicolet impact 410 FTIR spectrometer with KBr pellet in the 4000-400 cm^-1^ region, Both the excitation and emission pass width are 2.0 nm Hydrothermal reaction was performed in 15 mL Teflon-lined stainless steel autoclave.

Elemental analyses for C, H, N were performed on a Perkin-Elmer 2400 element analyzer. Powder X-ray diffraction measurements data were collected on a Rigaku D/max 2550 X-ray Powder Diffractometers. TGA was performed with a TGA Q500 V20.10 Build 36 which was collected from room temperature to 800 °C at a heating rate of 10 °C/min in a flowing N_2_ atmosphere. Magnetic susceptibility datas were obtained by SQUID magnetometer (Quantum MPMS) in the range of 2.0–300 K by using an applied field of 1000 Oe. Luminescent spectras were obtained on the FLS920 spectrofluorimeter.

**Experimental procedures**

Single crystals structure determination

The data collection and structural analysis were performed on a Rigaku RAXIS-RAPID equipped with a narrow-focus, 5.4 kW sealed tube X-ray source (graphite-monochromated Mo Kα radiation, λ = 0.71073 Å). The data were collected at a temperature of 20 ± 2 ˚C. The data processing was accomplished with the PROCESS-AUTO processing program. The structures were solved with the direct methods of SHELXL crystallographic software package and refined on *F*^2^ by full-matrx least square techniques. All non-hydrogen atoms of the compound was refined with anisotropic thermal parameters. All hydrogen atoms of the organic molecule were geometrical placed and added to the structure factor calculation. CCDC-1961577 contains the supplementary crystallographic data for this paper. The atomic coordinates for this structure has been deposited with the Cambridge Crystallographic Data Centre. The coordinates can be obtained, on request, from the Director, Cambridge Crystallographic Data Centre, 12 Union Road, Cambridge, CB2 1EZ, UK or at [www.ccdc.cam.ac.uk/conts/](http://www.ccdc.cam.ac.uk/conts/)retrieving.html. Basic information pertaining to crystal parameters and structure refinement is summarized in Table 1, and selected bond angles and distances is listed in Table S1 in the supporting information. As for the molecular formula of the synthesized compound 1, it is difficult to obtain the exact solvent molecules in the structure, so we further determined it using Platon/Squeeze, TGA, IR and Elemental analysis.

**Table S1**. Crystallographic data and structure refinement summary for compound 1

| Compound | **1** |
| --- | --- |
| Molecular formula | C_71_ H_67_ Co_3_ N_9_O_18_ |
| Formula weight | 1510.79 |
| Crystal system | Monoclinic |
| Space group | *C 2/c* |
| *a*, Å | 13.7148(9) |
| *Β,* Å | 21.0489(13) |
| *c*, Å | 22.8914(14) |
| *V*, Å^3^ | 6605.7(7) |
| *Z* | 4 |
| *D_calc_*, g/cm^3^ | 1.245 |
| *F*(000) | 2524.0 |
| *GoF* | 1.162 |
| *R*_1_, *wR*_2_ [I > 2σ(I)]^a,b^ | *R*_1_ = 0.0672,  *wR*_2_ = 0.1581 |
| *R*_1_, *wR*_2_ (all data) | *R*_1_ = 0.0344 *wR*_2_ = 0.0799 |
| ^a^*R*_1_ = ||*F*_o_| - |*F*_c_||/Σ|*F*|_o_. ^b^ wR_2_ = [Σ*w*(*F*_o_^2^ - *F*_c_^2^)2/Σ*w*(*F*_o_^2^)^2^]^1/2^. *w* =1/[σ^2^(*F*_o_^2^) + (*ap*)^2^ + (*bp*)], *p* = [max(*F*_o_^2^ or 0) + 2(*F*_c_^2^)]/3. | |

**Table S2. Selected bond distances (Å) and angles (°) for compound 1**

| Co1-O1 | 2.0074(11) | Co2-O2^iii^ | 2.0526(11) |
| --- | --- | --- | --- |
| Co1-N2 | 2.0855(15) | Co2-O2 | 2.0526(11) |
| Co1-O6^i^ | 2.093(11) | Co2-O6^iv^ | 2.0982(11) |
| Co1-N1 | 2.115(14) | Co2-O6^i^ | 2.0982(11) |
| Co1-O4^ii^ | 2.1825(14) | Co2-O3^v^ | 2.1080(12) |
| Co1-O3^ii^ | 2.2725(14) | Co2-O3^ii^ | 2.1080(12) |
| O1-Co1-N2 | 94.91(5) | O2^iii^-Co2-O2 | 180.0 |
| O1-Co1-O6^i^ | 99.06(5) | O2^iii^-Co2-O6^iv^ | 88.09(5) |
| O6^i^-Co1-N2 | 118.24(5) | O2-Co2-O6^iv^ | 91.91(4) |
| O1-Co1-N1 | 171.34(5) | O2^iii^-Co2-O6^i^ | 91.91(5) |
| N2-Co1-N1 | 76.79(6) | O2-Co2-O6^i^ | 88.09(5) |
| N1-Co1-O6^i^ | 83.00(5) | O6^iv^-Co2-O6^i^ | 180.00(6) |
| O4^ii^-Co1-O1 | 97.18(5) | O2^iii^-Co2-O3^v^ | 93.68(5) |
| N2-Co1-O4^ii^ | 104.59(5) | O2-Co2-O3^v^ | 86.32(5) |
| O4^ii^-Co1-O6^i^ | 132.26(5) | O6^iv^-Co2-O3^v^ | 80.11(5) |
| N1-Co1-O4^ii^ | 87.44(6) | O6^i^-Co2-O3^v^ | 99.89(5) |
| O1-Co1-O3^ii^ | 91.54(5) | O2^iii^-Co2-O3^ii^ | 86.32(5) |
| N2-Co1-O3^ii^ | 162.54(5) | O2-Co2-O3^ii^ | 93.68(5) |
| O6^i^-Co1-O3^ii^ | 76.52(4) | O6^iv^-Co2-O3^ii^ | 99.89(5) |
| N1-Co1-O3^ii^ | 97.12(5) | O6^i^-Co2-O3^ii^ | 80.11(5) |
| O4^ii^-Co1-O3^ii^ | 58.42(5) | O3^v^-Co2-O3^ii^ | 180.0 |

Symmetry codes: (i) *x*−1, *y*, *z*; (ii) *x*−1/2, −*y*+1/2, *z*−1/2; (iii) −*x*+1/2, −*y*+1/2, −*z*+1; (iv) −*x*+3/2, −*y*+1/2, −*z*+1; (v) −*x*+1, *y*, −*z*+3/2; (vi) *x*+1/2, −*y*+1/2, *z*+1/2; (vii) *x*+1, *y*, *z*.

**Table S3**. CO_2_ uptake of different MOFs at 298 K

| MOFs | **298 K (cm^3^**•g-^1^) |
| --- | --- |
| [(Zn_4_O)_2_(PDDA)_6_(H_2_O)_2_]·10DMF | 31 |
| JUC-MOF56 | 38 |
| {[Cd_2_(tdz)_2_(4,4’-bpy)_2_]•6.5H_2_O}n | 29.12 |
| [Zn_2_(TCA)(BIB)_2.5_]•(NO_3_) | 48.4 |
| ([Zr_6_O_4_(OH)_8_(H_2_O)_4_(BTEB)_2_] | 42 |
| {[Cd_4_(hbhdpy)_2_(bdc-NH_2_)_3_ (DMA)_2_]•(H_2_O)_4_}n | 30 |


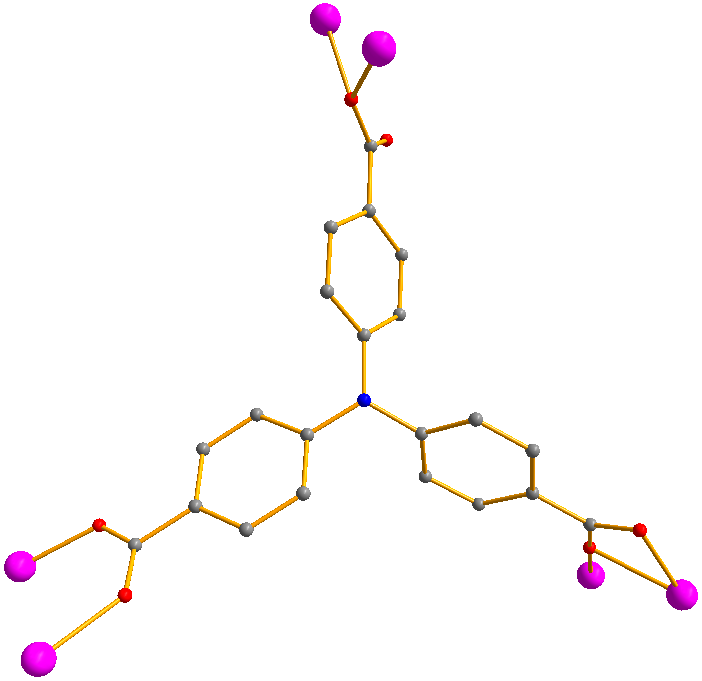


**Scheme 1** The coordination mode of L^3-^ (C: Gray, O: Red, N: Blue, Co:Purple )


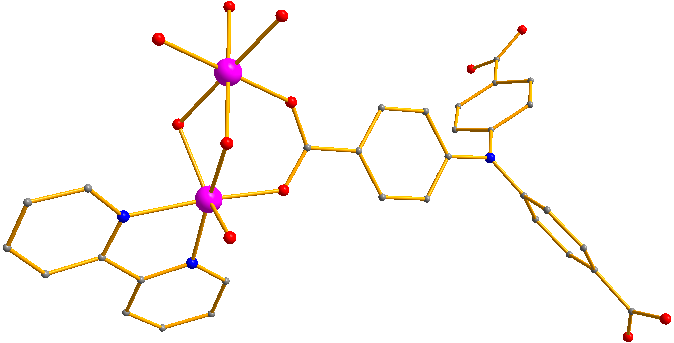


**Fig. S1** The asymmetric unit of compound 1 (C: Gray, O: Red, N: Blue, Co:Purple )


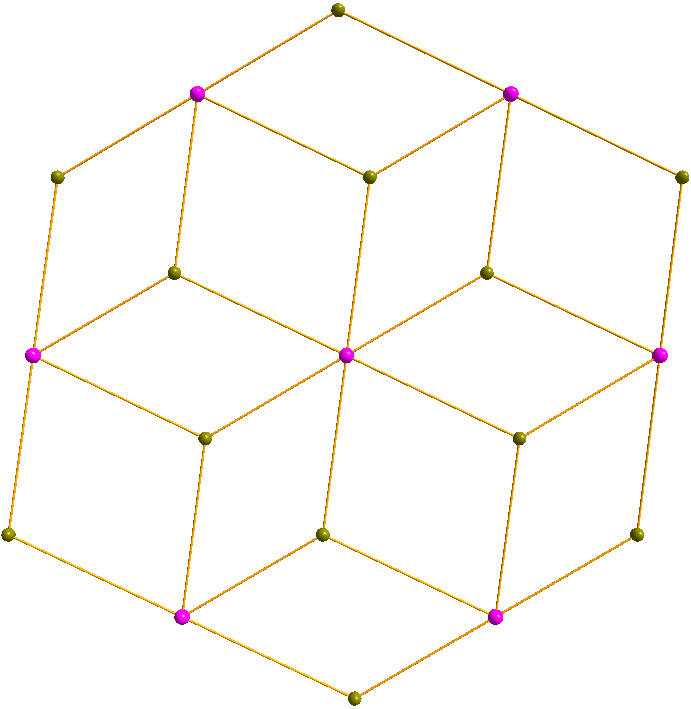


**Fig.S2** The topology of compound 1


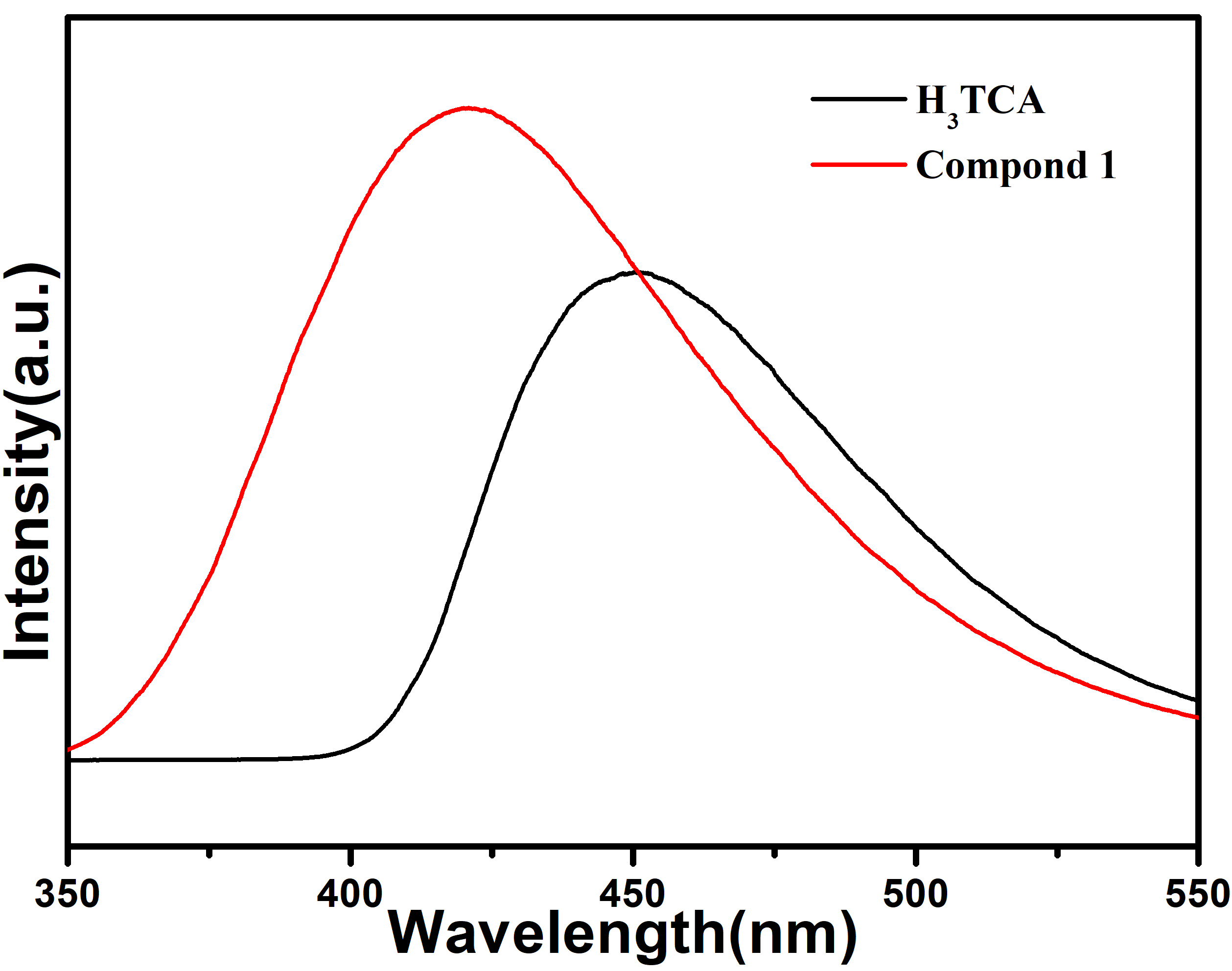


**Fig. S3** Fluorescent spectra of H_3_L and Compound 1

**
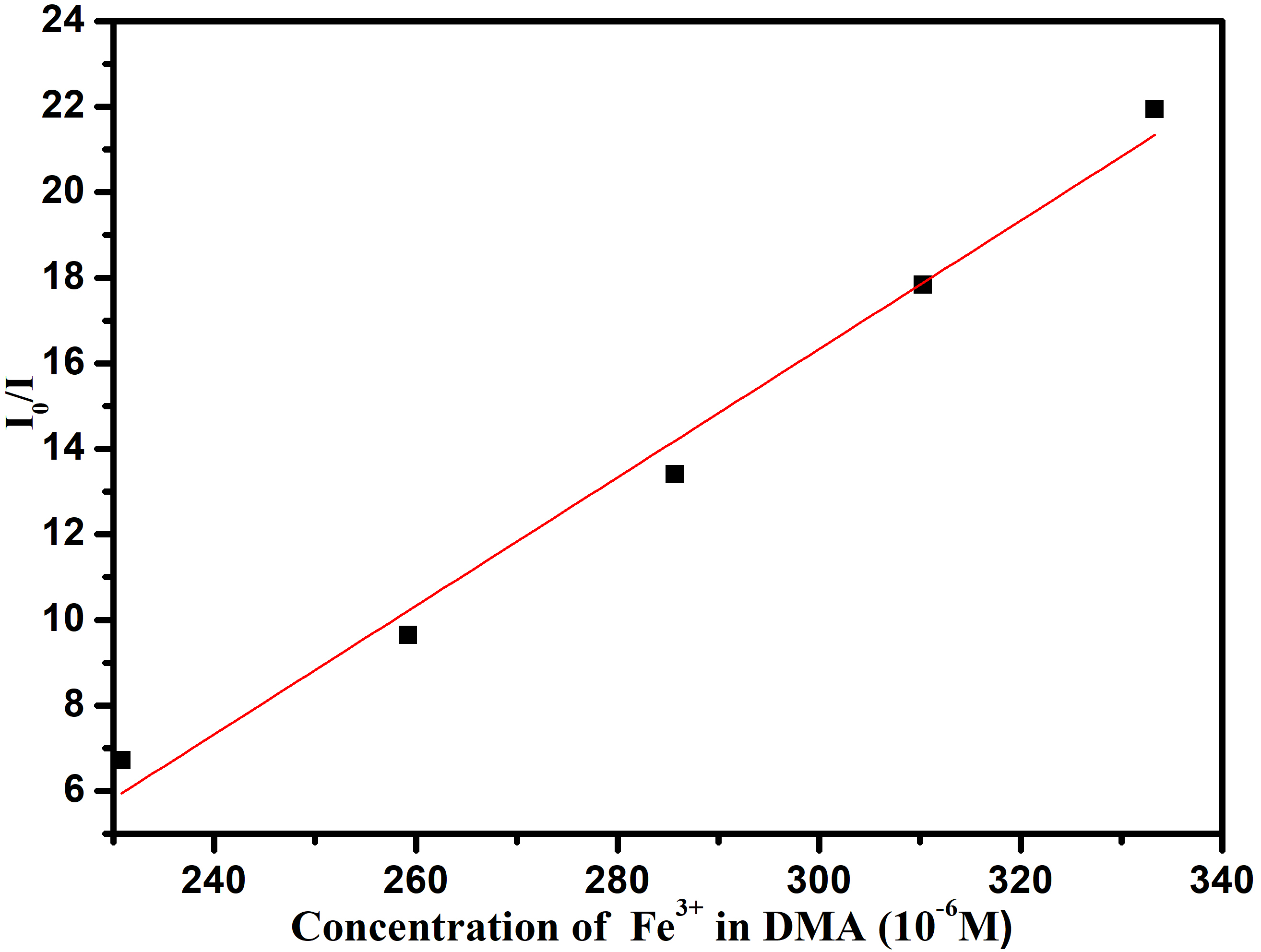
**

**Fig. S4** Stern-Volmer plots of I_0_/I vs. the concentration of Fe^3+^ of compound 1


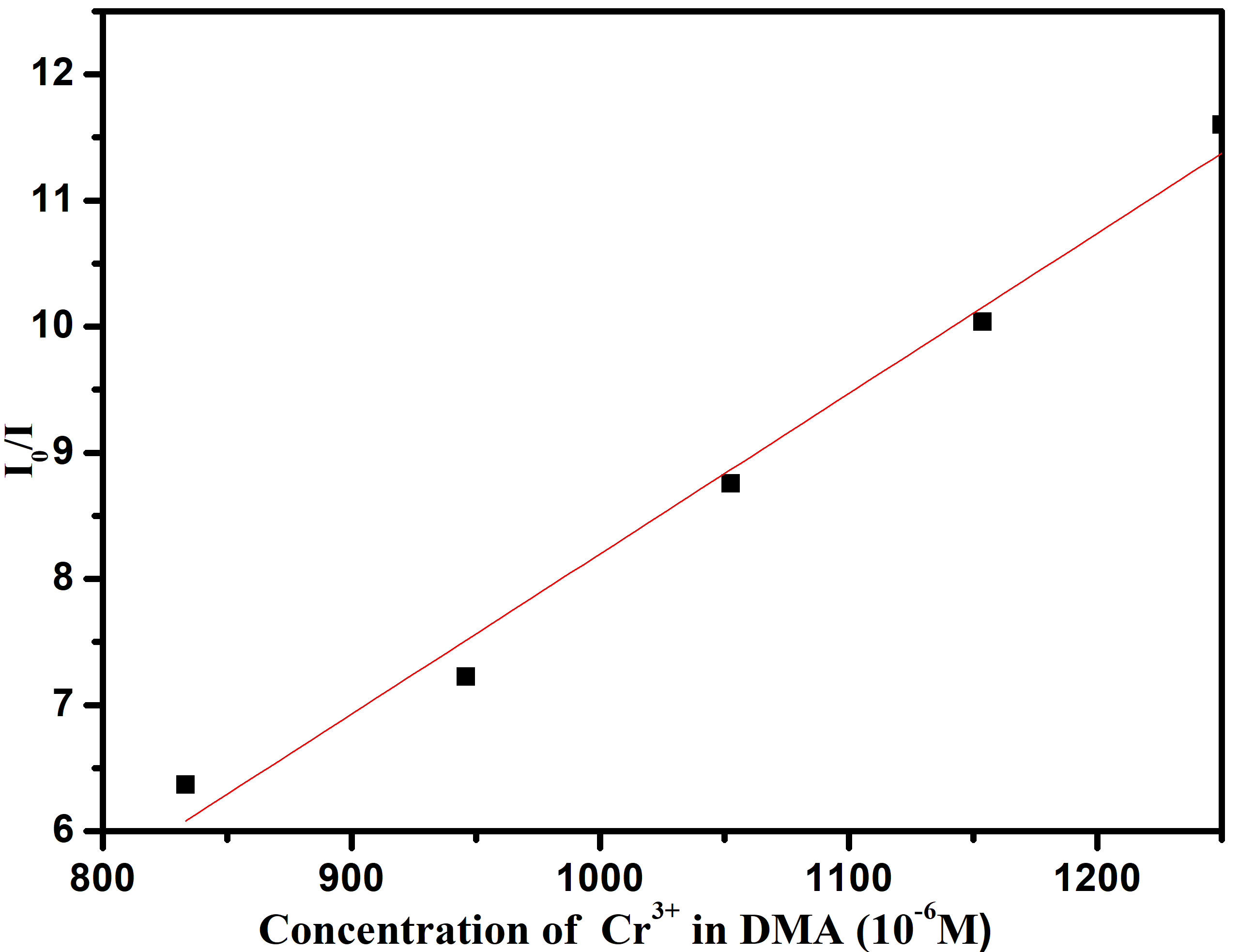


**Fig. S5** Stern-Volmer plots of I_0_/I vs. the concentration of Cr^3+^ of compound 1


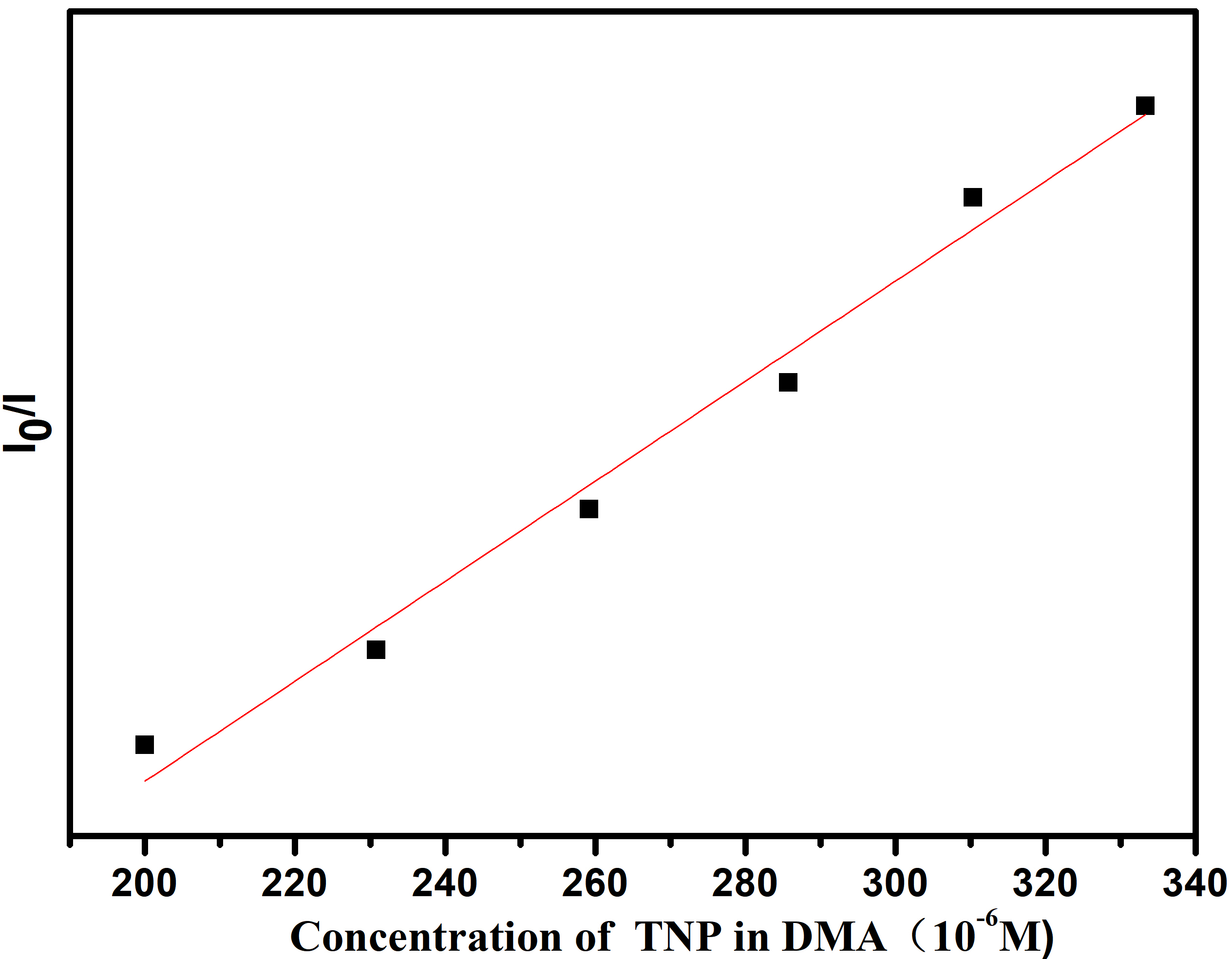


**Fig. S6** Stern-Volmer plots of I_0_/I vs. the concentration of TNP of compound 1


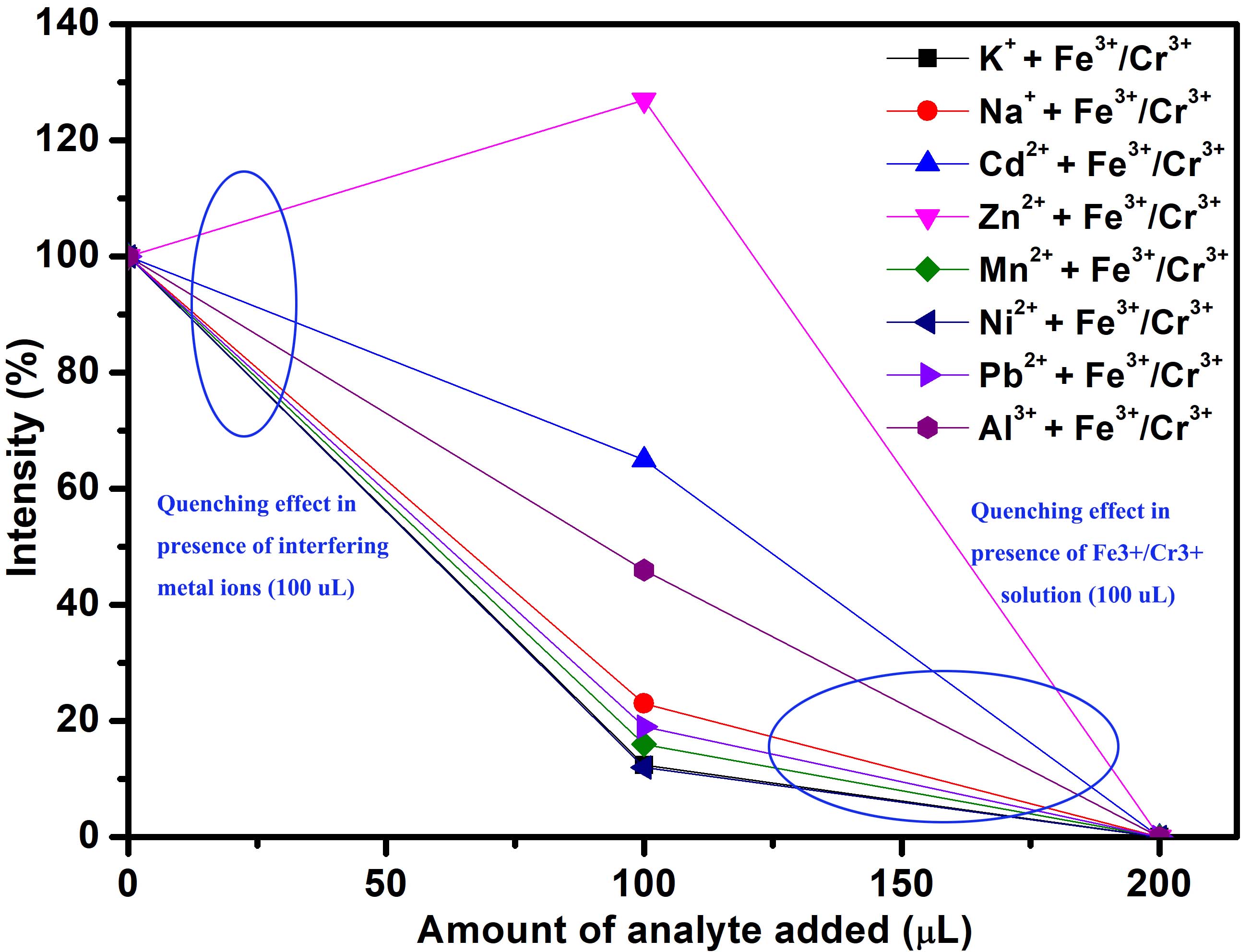


**Fig. S7**  The anti-interference experiments for Fe^3+^ and Cr^3+^ (10^-2^ M)


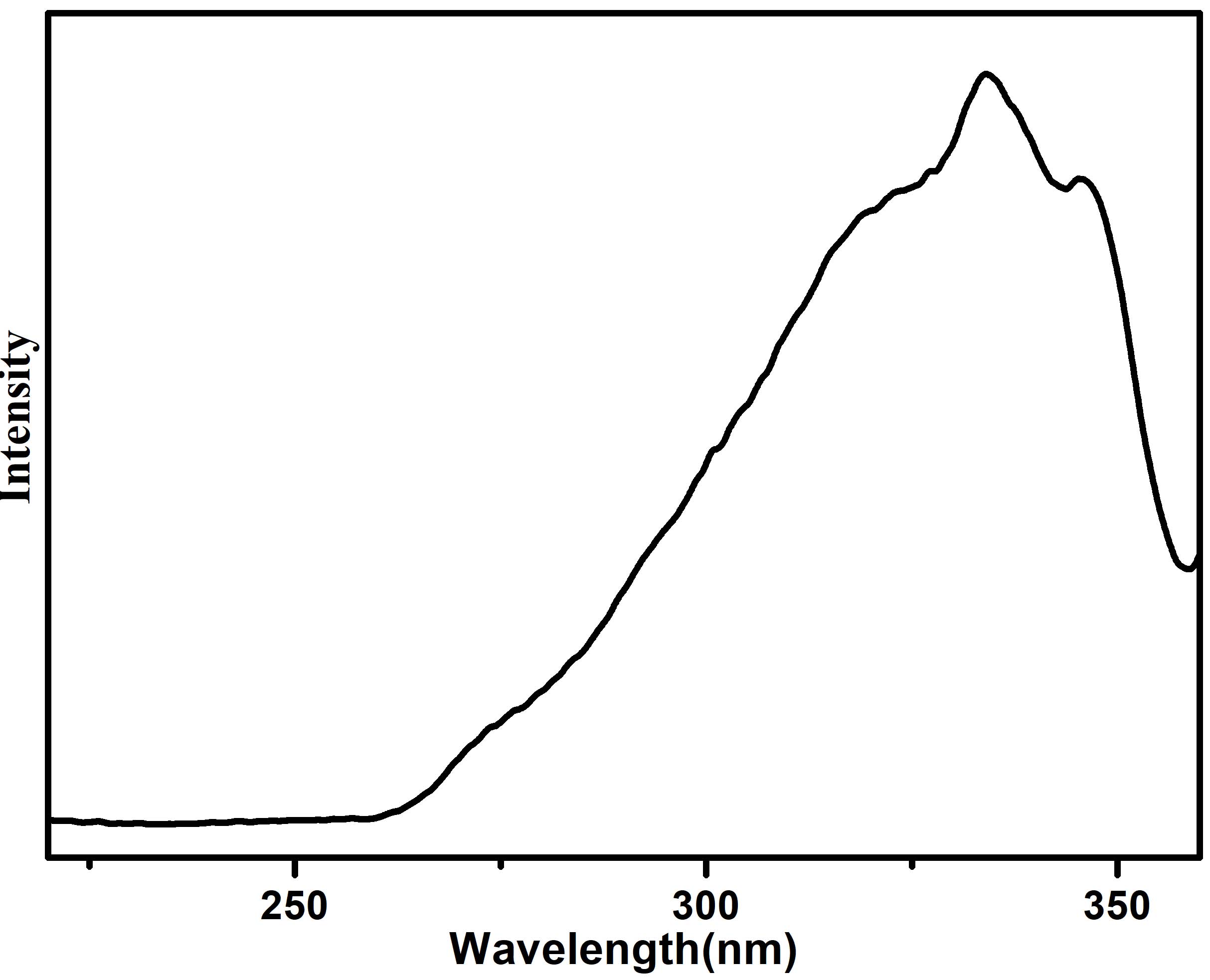


**Fig. S8**  The excitation of compound 1a


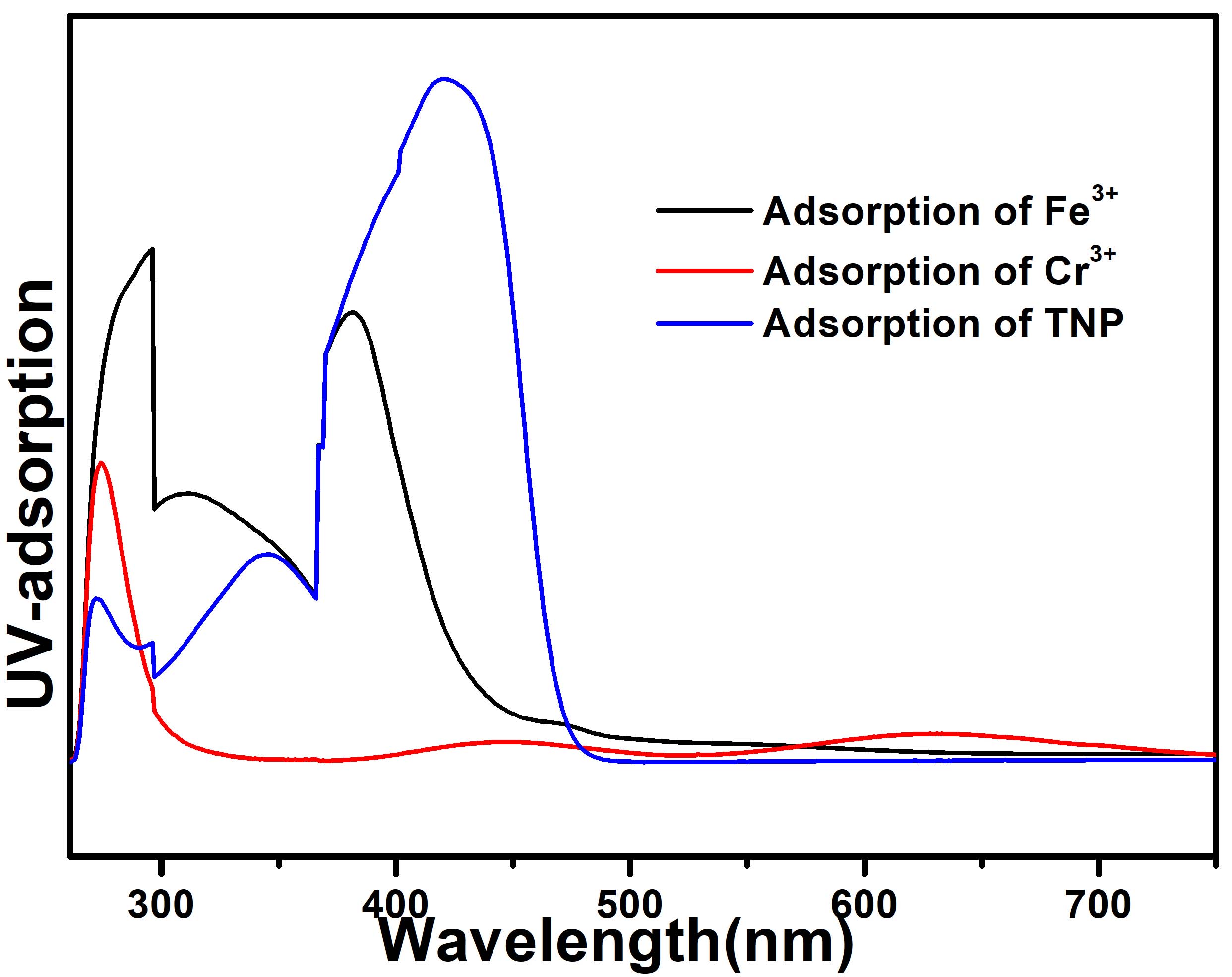


**Fig. S9** The UV adsorption of Fe^3+^, Cr^3+^ and TNP


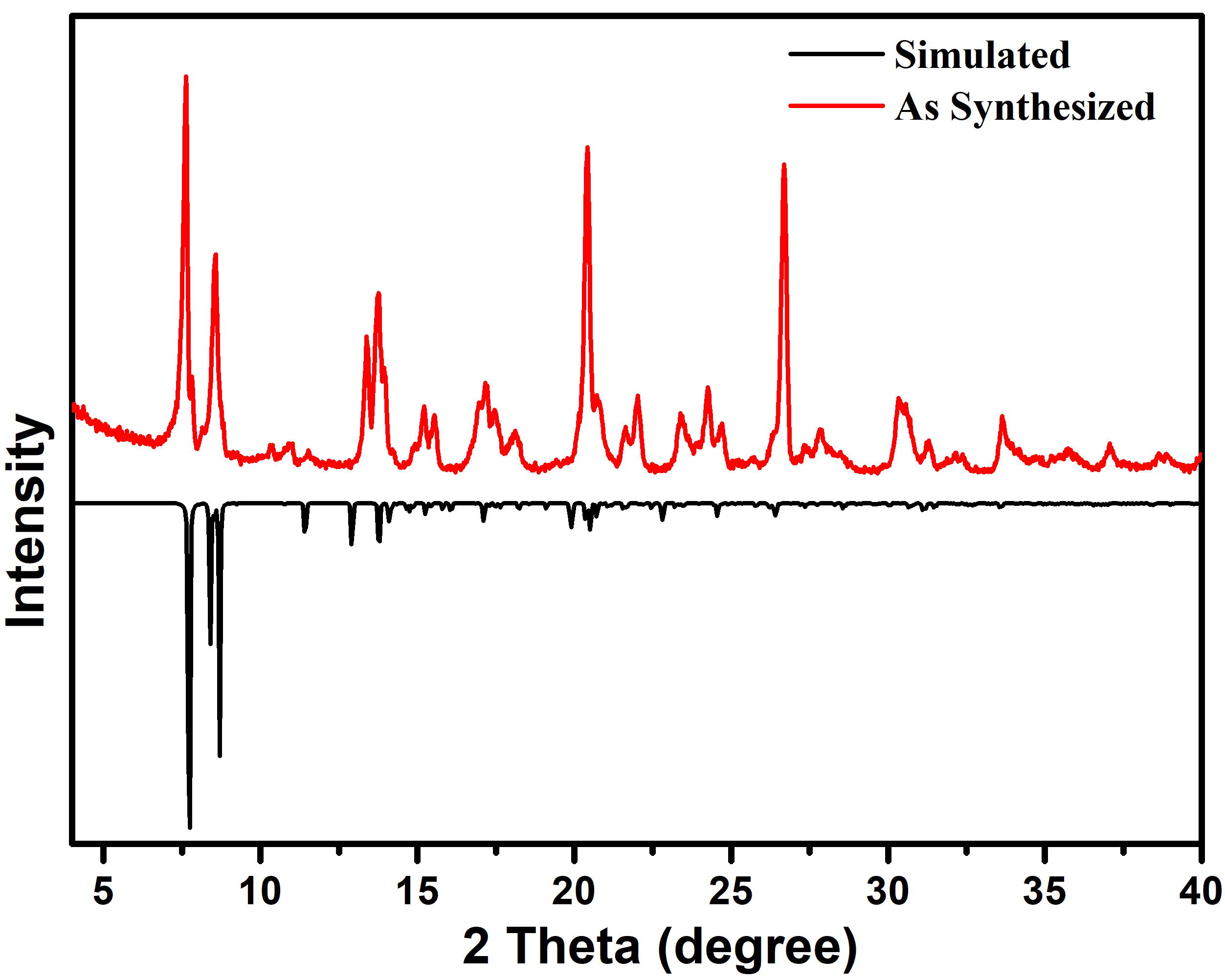


**Fig. S10** The PXRD of compound **1**


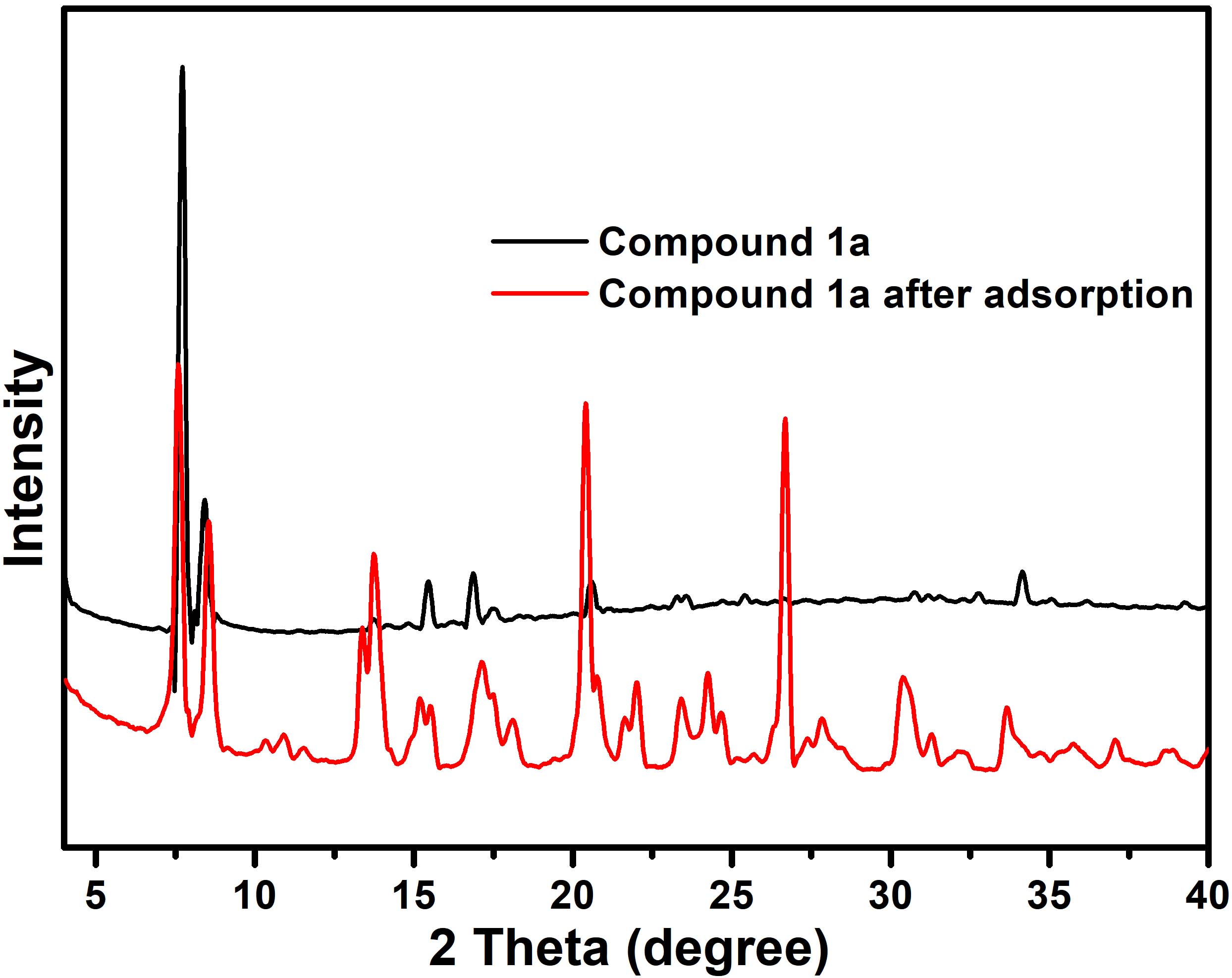


**Fig. S11** The PXRD of compound 1a and after the adsorption


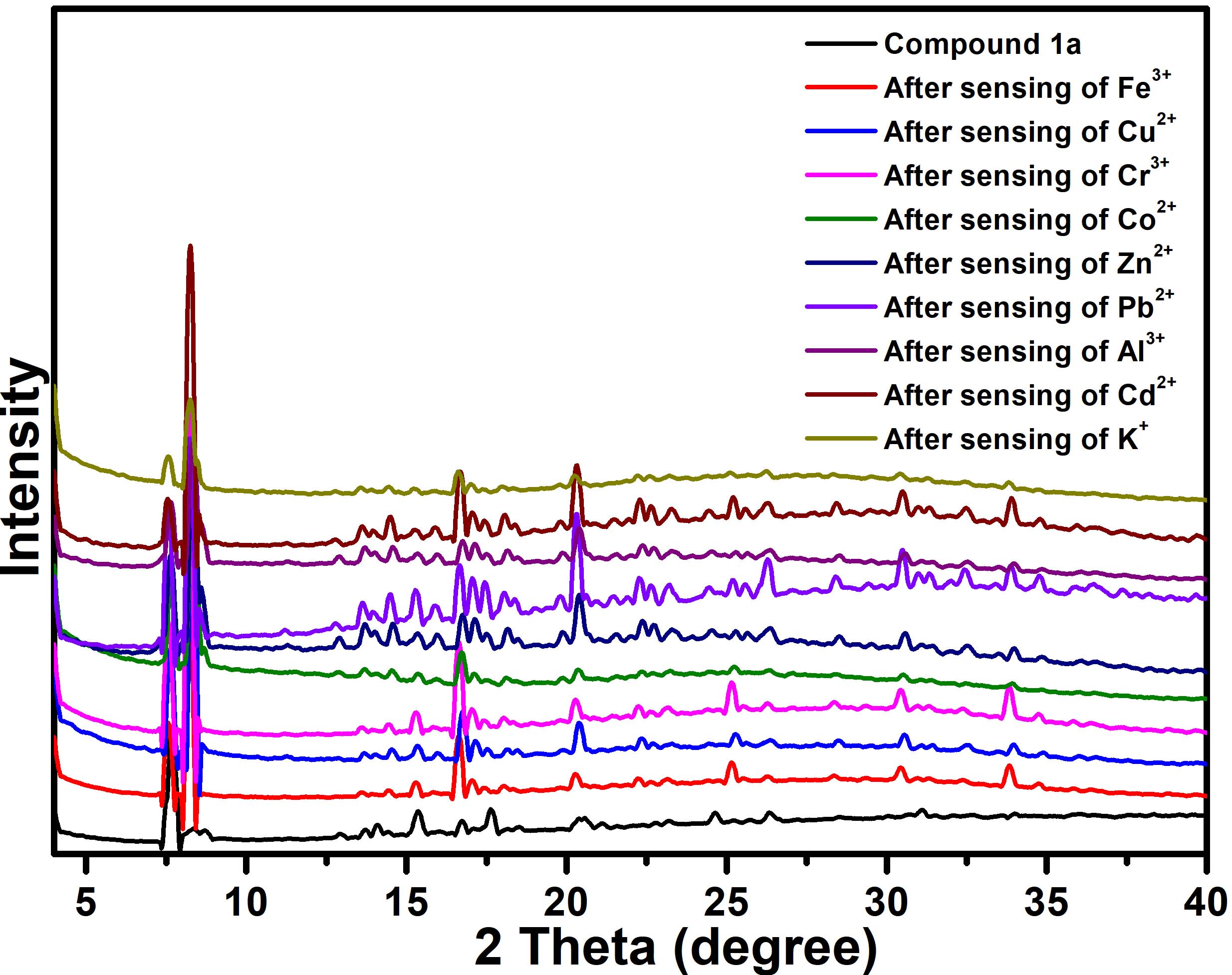


**Fig. S12** The PXRD of compound 1a and after sensing of the metal ions


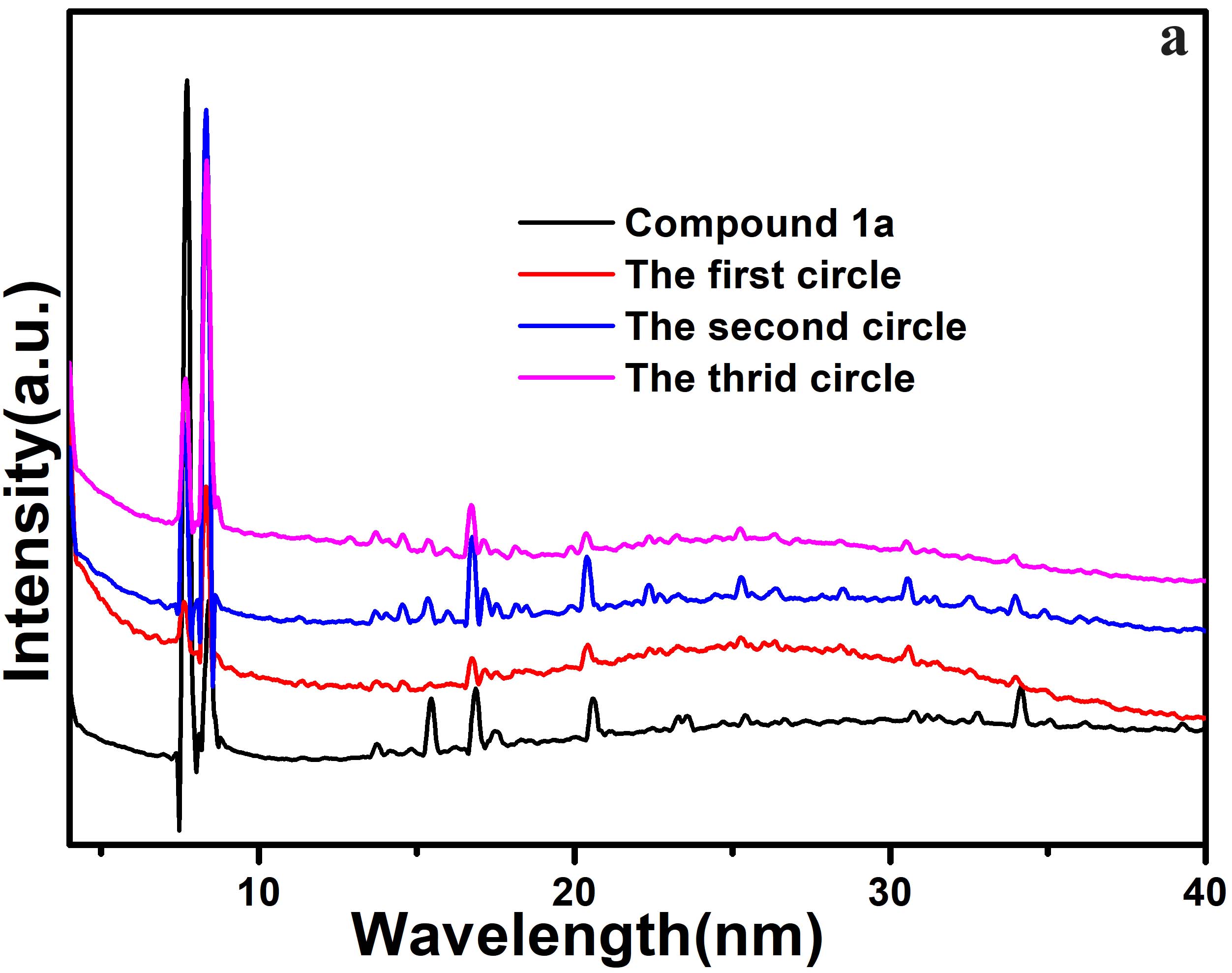


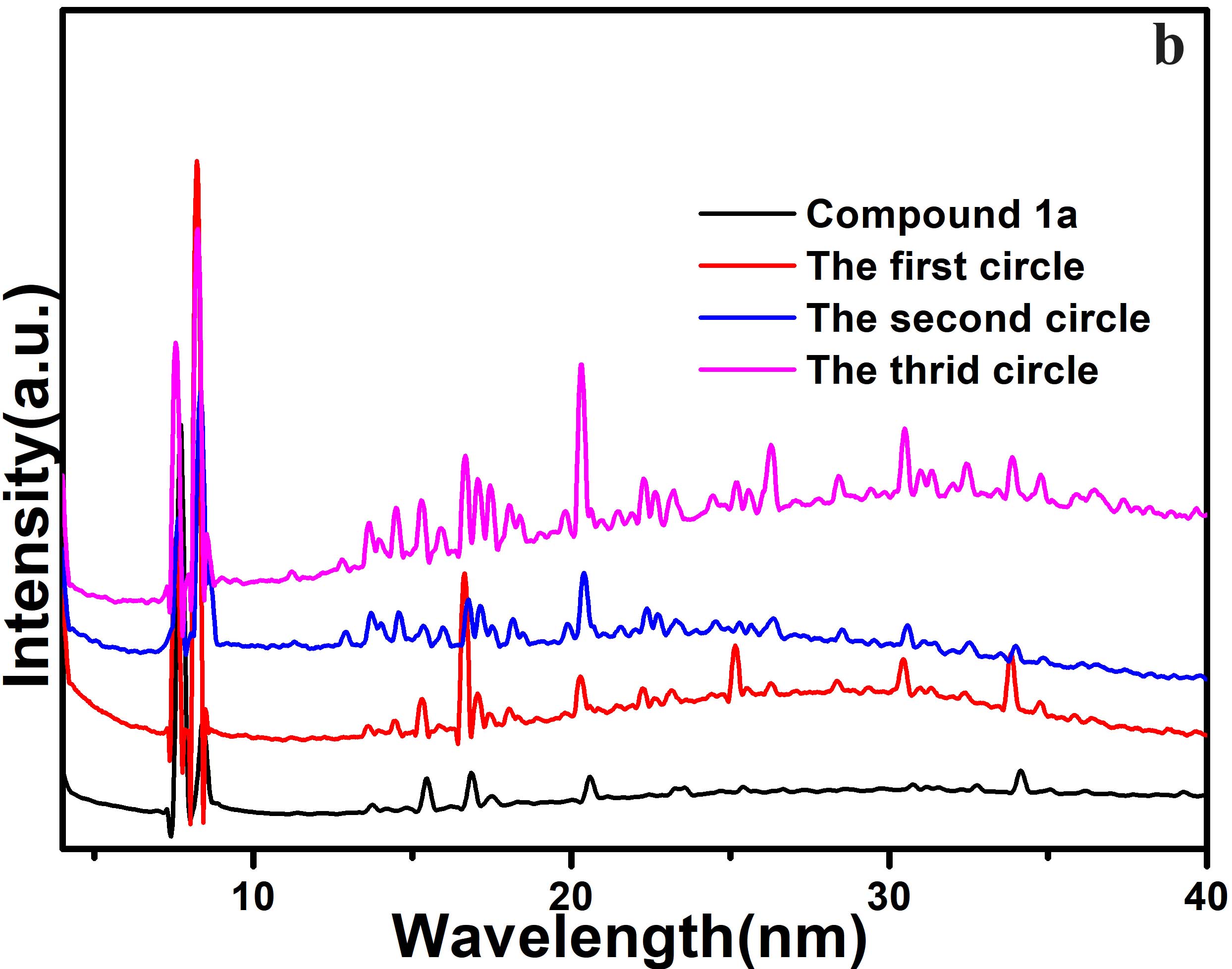


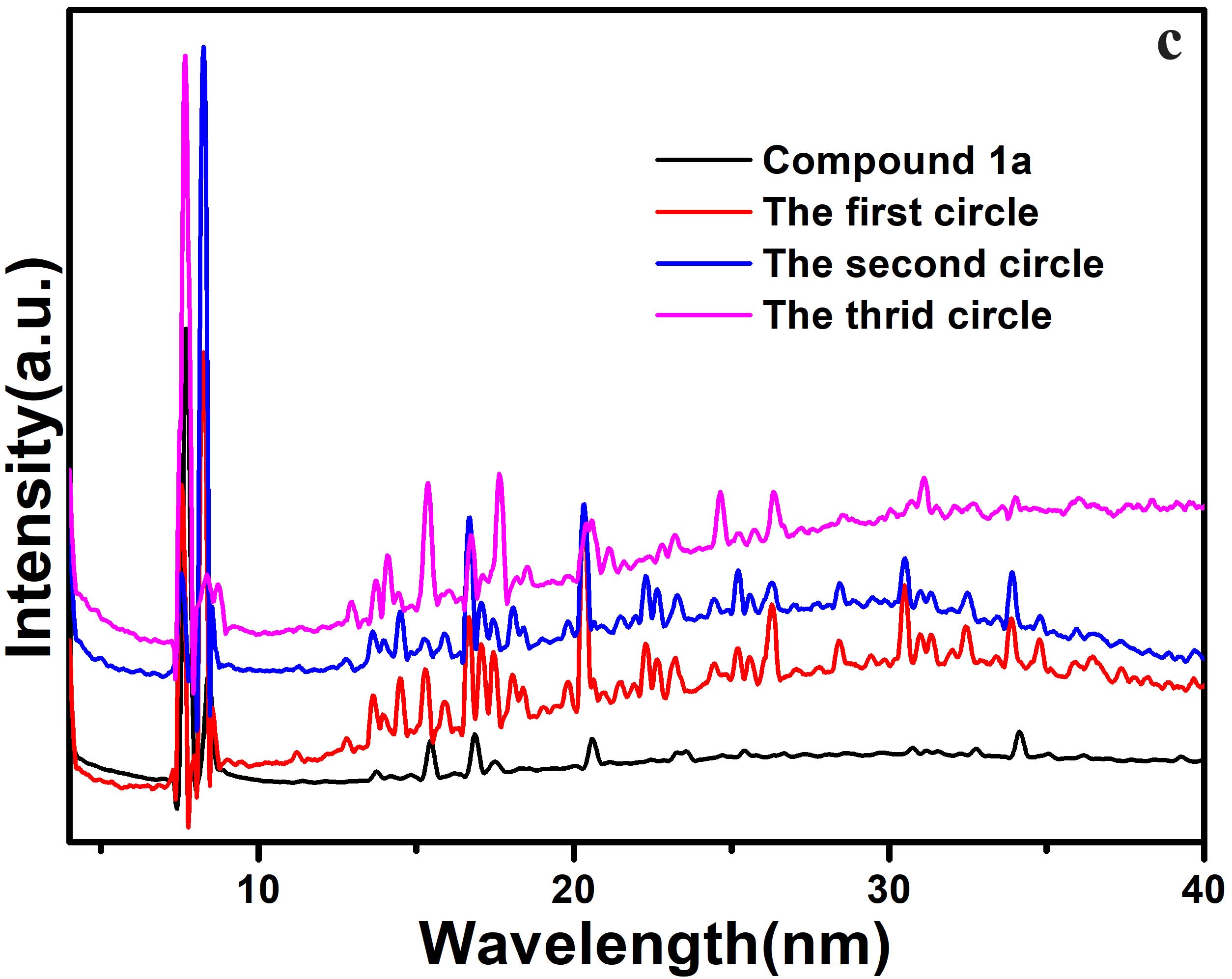


Fig. S13 a) Three circle PXRD experiments of compound 1a after sensing Fe^3+^; b) Three circle PXRD experiments of compound 1a after sensing Cr^3+^; c) Three circle PXRD experiments of compound 1a after sensing TNP


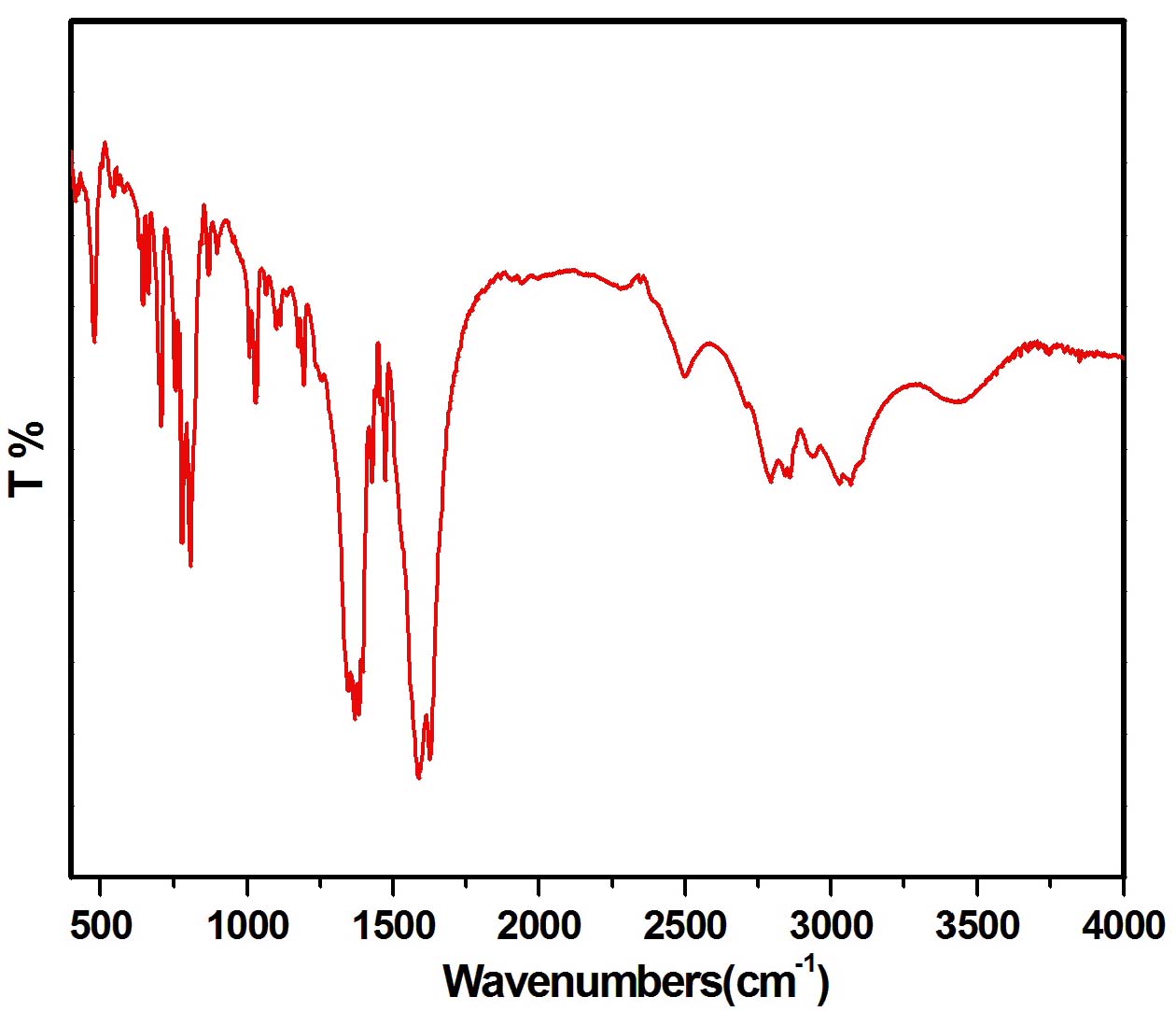


**Fig. S14** The IR spectrum of compound **1**


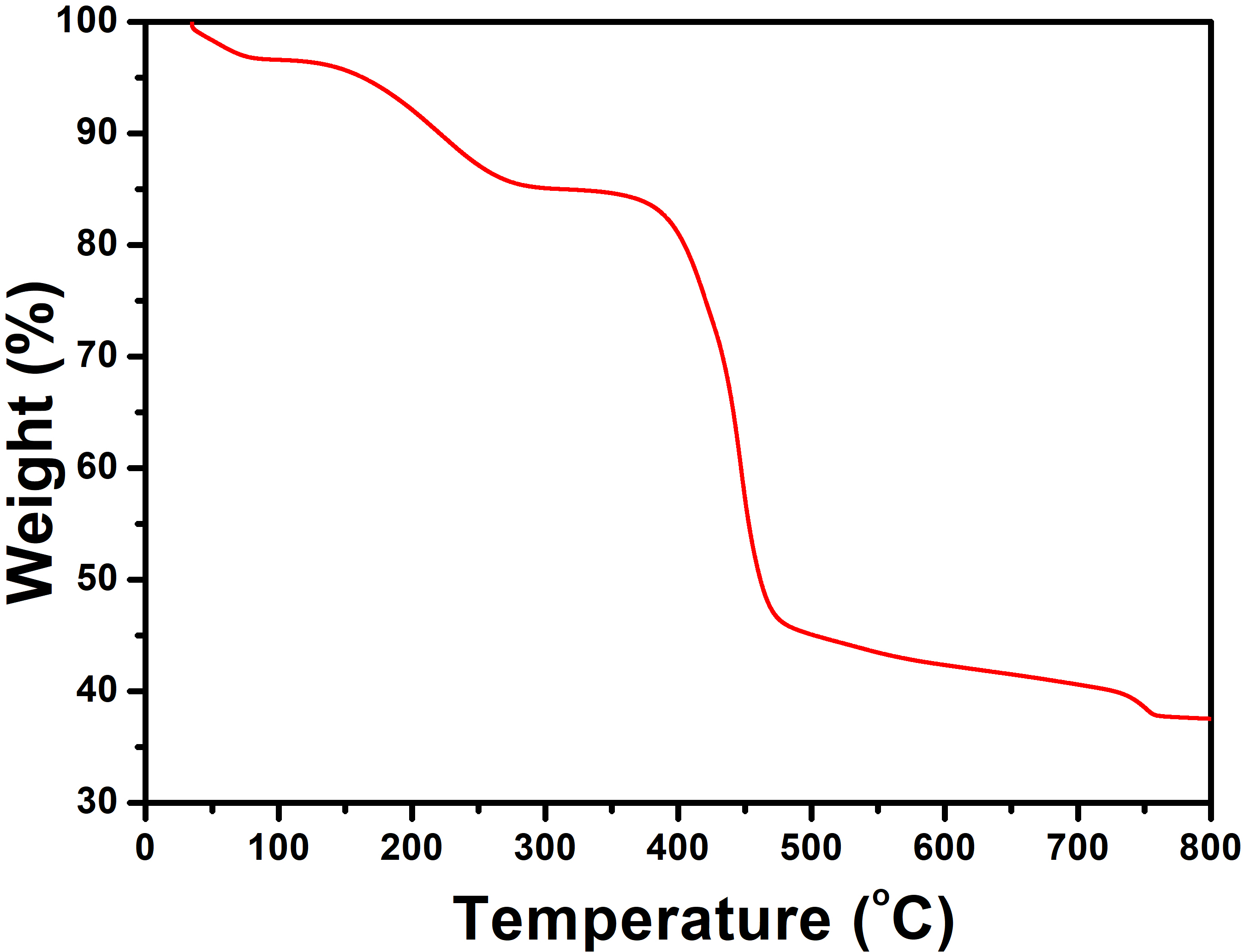


**Fig. S15** TGA of compound **1**


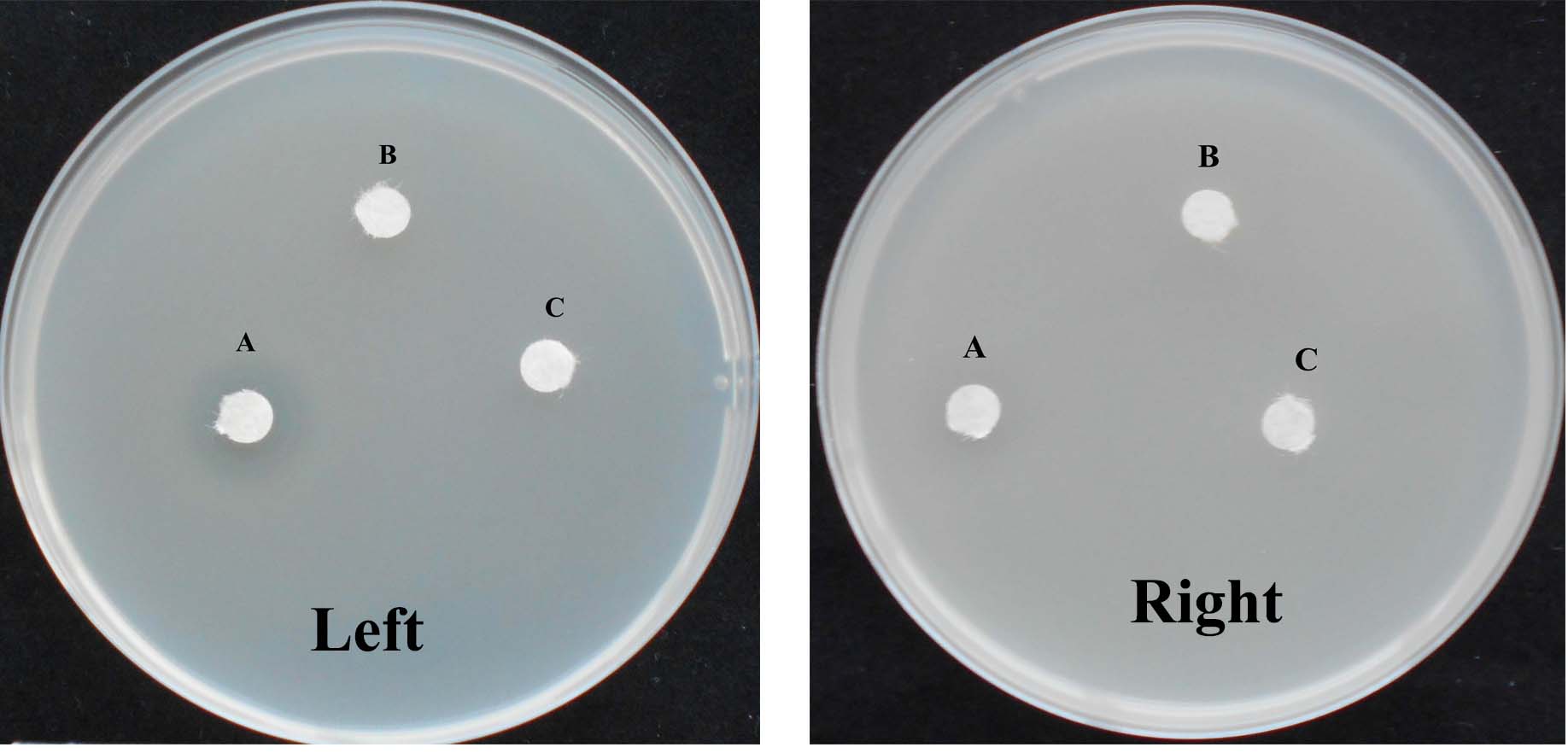


**Fig. S16**  Left:The inhibition zone test graphs of compound 1 compound 1(A)、H_3_L(B)、2,2-bipy (C) against E. coli respectively; Right: The inhibition zone test graphs of compound 1(A)、H_3_L(B)、2,2-bipy (C) against S. aureus respectively
